# Exploring 2D Graphene‐Based Nanomaterials for Biomedical Applications: A Theoretical Modeling Perspective

**DOI:** 10.1002/smsc.202400505

**Published:** 2025-03-16

**Authors:** Alexa Kamboukos, Nevena Todorova, Irene Yarovsky

**Affiliations:** ^1^ School of Engineering RMIT University Melbourne Victoria 3001 Australia

**Keywords:** biomedicine, graphene nanomaterials, graphene oxide, molecular simulations, nanotoxicity

## Abstract

Two‐dimensional (2D) graphene‐based nanomaterials (GNMs) have shown potential in biomedical applications, including diagnostics, therapeutics, and drug delivery, due to their unique combination of properties such as mechanical strength, excellent electrical and thermal conductivity as well as high adsorption capacity which, combined with the ease of their surface functionalization, enable biocompatibility and bioactivity. Theoretical molecular modeling can advance our understanding of the biomedical potential of 2D graphene‐based nanomaterials by providing insights into the structure, dynamics, and interactions of these nanomaterials with biological systems, at the level of detail that experiments alone cannot currently access. This perspective highlights recent computational modeling advances and challenges in examining the interactions of 2D graphene‐based nanomaterials with physiologically relevant biomolecular systems, including aqueous solutions, peptides, proteins, nucleic acids, lipid membranes, and pharmaceutical drug molecules. Examples of the theoretical contributions to design of graphene‐based biomaterials and devices are also provided.

## Graphical abstract

Two‐dimensional graphene‐based nanomaterials are of significant interest in biomedical applications due to their unique physicochemical properties. Computational modeling provides useful insights into their structure and interactions with biological systems, aiding the nanoparticle design. The successes and challenges of theoretical simulations in exploring the interactions of graphene‐based nanomaterials with physiologically relevant environments are reviewed.

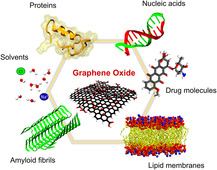

## 1 Introduction

Owing to their small size (<100 nm) and large surface‐to‐volume ratio, nanomaterials (NMs) exhibit unique physicochemical properties, making them promising candidates for biomedical applications including therapeutics, diagnostics, tissue engineering, and drug delivery.^[^ ^1^, ^2^ ^]^ Within the broad category of NMs, inorganic carbon‐based NMs encompass many derivatives varying in their dimension, shape, curvature, and chemical functionalization. These include carbon nanotubes (CNTs), graphene quantum dots (GQDs), fullerenes (C60 and others), pristine graphene (PG), graphene oxide (GO), and reduced graphene oxide (rGO). Two‐dimensional (2D) graphene‐based NMs (GNMs) such as graphene and its derivatives GO and rGO, have shown great potential in biomedical applications owing to their biocompatibility, mechanical strength, electrical and thermal conductivity, and ease of surface functionalization.^[^ ^3^, ^4^, ^5^ ^]^ These graphene‐based NMs have the capacity to interact with a variety of biomolecules via specific and nonspecific interactions, driven by their heterogeneous surface properties.

**Figure 1:**
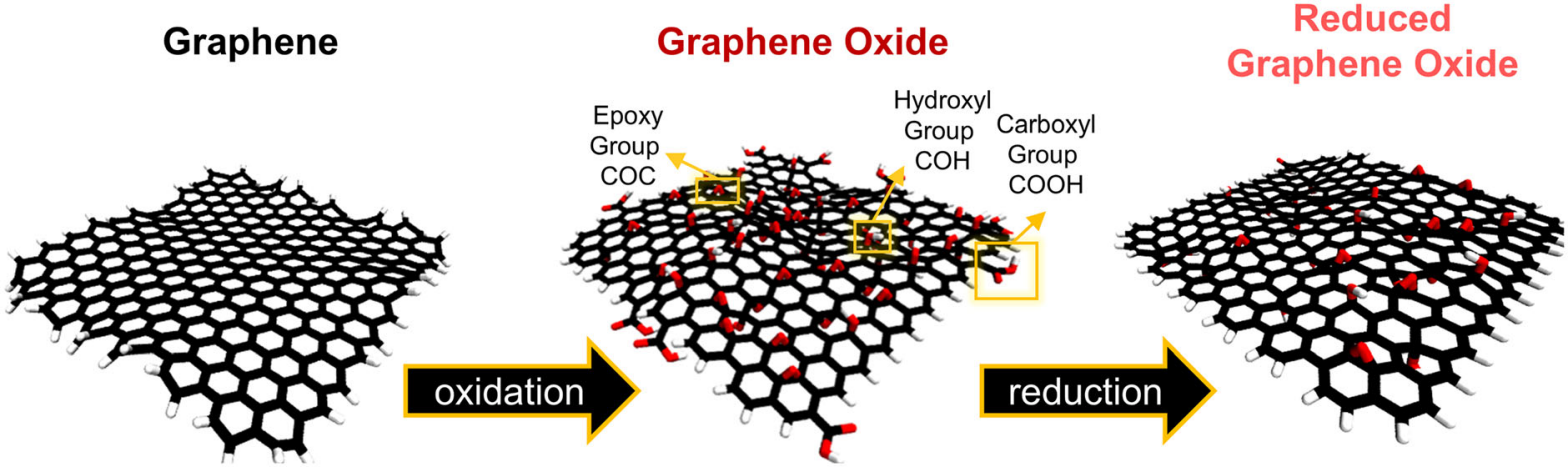
Typical structure of 2D graphene‐based NMs. Graphene‐based nanoflake (NF) models were created using the VMD^[^ ^480^ ^]^ software and modeled by explicit solvent molecular dynamics. (MD) simulations.^[^ ^218^ ^]^

Graphene consists of a single layer of covalently bonded aromatic *sp* ^2^ hybridized carbon (**Figure** **1**). While PG exhibits many appealing physical and chemical properties for applications in materials science and nanotechnology, the hydrophobic nature limits graphene applications in biomedicine due to inherent incompatibility with physiological (aqueous) environment. However, the surface properties of graphene can easily be tuned to create functionalized graphene derivatives targeting bioapplications, i.e., a hydrophilic graphene derivative, known as GO, is commonly obtained via surface oxidation. A typical GO structure can comprise a variety of covalently bonded oxygen‐containing functional groups, including epoxy, hydroxyl, carbonyl, and carboxyl groups (Figure 1). The oxygen‐containing functional groups enhance the NM's dispersibility, solubility, and biocompatibility in biological environments, making oxidized GNMs promising materials for targeted biomedical applications. The Lerf–Klinowski model^[^ ^6^ ^]^ is the most widely used structural model of GO nanoparticles, which assumes all oxygen‐containing functional groups are incorporated within graphene's basal plane as well as covalently attached to the edges. The surface oxygen concentration of a GO material can be tuned through surface reduction, producing more hydrophobic GO derivates known as rGO (Figure 1). The ability to tailor the oxygen concentration and distribution within the GO materials has led to research specifically focusing on optimizing the surface properties of GO for effective use in biomedicine.^[^ ^7^, ^8^, ^9^ ^]^ Common functionalization strategies include covalent and noncovalent binding of small molecules, peptides, DNA fragments, proteins (incl. enzymes), polymers as well as inorganic molecules, such as quantum dots and nanoparticles, to GNMs.^[^ ^10^, ^11^ ^]^ Besides graphene and GO derivatives, various other 2D graphene analogs exist, including but not limited to chemically doped graphene, graphyne, graphdiyne, and penta‐graphene. For example, graphynes consisting of both *sp* and *sp* ^2^ hybridized carbon bonds can take many structural forms, including graphyne, graphdiyne, and graphyne‐*n*,^[^ ^12^ ^]^ and penta‐graphene is comprised of carbon pentagons,^[^ ^13^ ^]^ however, these are not in the scope of this review.

The surface of 2D GNMs can provide a template for the adsorption and release of various solvated biological molecules, including peptides, proteins, DNA fragments, and lipids, which introduces an opportunity for tailoring the biomolecule–GNM interactions for targeted biomedical applications, as recently summarized in detail in several reviews.^[^ ^2^, ^14^, ^15^, ^16^, ^17^, ^18^, ^19^, ^20^ ^]^ However, safe and effective applications of 2D GNMs in biomedicine necessitate a comprehensive understanding of their effects on the biological environment, as toxicity of 2D GNMs remains a challenge.^[^ ^21^, ^22^, ^23^, ^24^, ^25^, ^26^ ^]^ Experimental studies have shown toxicity levels of GNMs vary depending on their preparation method, size, concentration, and functionalization.^[^ ^27^ ^]^ One of the main GNM advantages also represents an issue for their widespread biomedical applications, namely, their high biological reactivity, which makes it difficult to control their interactions with the biological medium, including in the cellular environment. The uncontrolled GNM interactions can lead to undesirable, and often, unanticipated consequences, such as the formation of biomolecular coronas^[^ ^28^, ^29^ ^]^ which can impart new biological identify to the nanoparticle and lead to its unintended interactions with the biological environment. The complexity of intended and unintended interactions of GNMs for biomedical applications warrants the need for fundamental understanding of the physicochemical mechanisms underlying the 2D GNMs interactions with biological molecules in realistic physiological environments.

**Figure 2:**
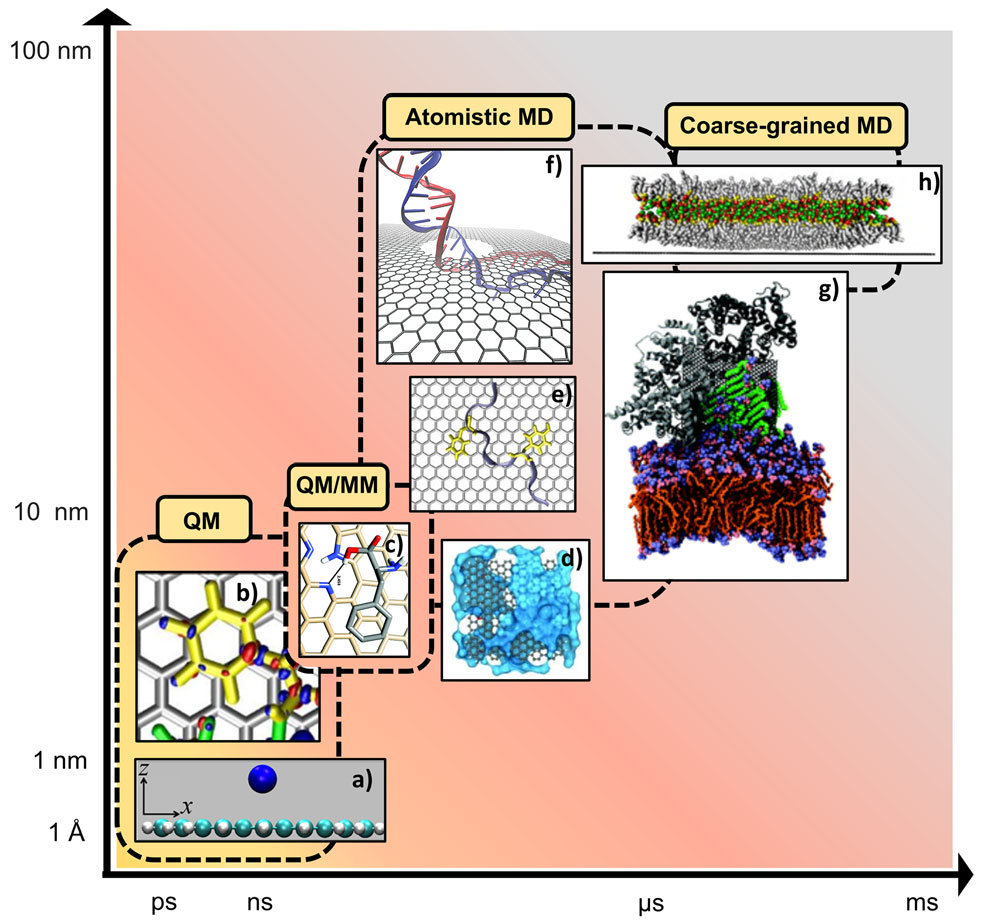
Schematic showing relative time and length scales for modeling interactions between 2D graphene‐based NMs and biomedically important molecules using QM, classical MD and hybrid QM/MM methods, illustrated by studies of: a) PG and ions^[^ ^252^ ^]^ b,e) PG and apolipoprotein C‐II peptide fragment 60–70,^[^ ^68^ ^]^ c) nitrogen‐doped graphene and phenylalanine amino acid (AA),^[^ ^481^ ^]^ d) rGO and water,^[^ ^218^ ^]^ f) graphene nanopore and double‐stranded DNA,^[^ ^112^ ^]^ g) PG and a lipid bilayer,^[^ ^69^ ^]^ h) PG and an inverted bilayer.^[^ ^167^ ^]^ (a) Adapted with permission.^[^ ^252^ ^]^ Copyright 2017, American Chemical Society. (b,e) Adapted under terms of the CC‐BY 3.0 license.^[^ ^68^ ^]^ Copyright 2013, The Authors. Published by PLoS. (c) Reproduced with permission.^[^ ^481^ ^]^ Copyright 2016, The Royal Society of Chemistry. (d) Adapted with permission,^[^ ^218^ ^]^ https://pubs.acs.org/doi/10.1021/acsomega.8b00866. Copyright 2018, American Chemical Society. Any further permissions related to this material should be directed to the American Chemical Society. (f) Adapted with permission.^[^ ^112^ ^]^ Copyright 2011, American Chemical Society. (g) Adapted with permission.^[^ ^69^ ^]^ Copyright 2015, The Royal Society of Chemistry. (h) Adapted under terms of the CC‐BY 4.0 license.^[^ ^167^ ^]^ Copyright 2017, The Authors. Published by American Chemical Society.

Theoretical molecular modeling enables the investigation of the structure, dynamics, and interactions between NMs and biological molecules at spatial and temporal resolution difficult or impossible to achieve by experiments alone (**Figure** **2**).^[^ ^30^, ^31^, ^32^ ^]^ Computational modeling approaches based on quantum mechanics (QM), including the wave function and density functional theory methods,^[^ ^33^ ^]^ are the most accurate but expensive methods to calculate the electronic structure of atoms and molecules. These techniques are primarily used to predict molecular structure, dynamics, electron densities, binding energies, and reaction pathways for relatively small‐scale system (<100 atoms) (Figure 2a,b). However, the high computational cost of these methods limits their applicability to larger systems.

Modeling of larger and more complex bio–nano systems requires the use of classical molecular mechanics (MM) approaches which employ interatomic forcefields (FFs) to approximate the potential energy of a molecular system as a function of atomic coordinates, where individual atoms or groups of atoms are modeled as optionally charged mechanically connected interacting particles. This simplified approach is useful for investigating structural, physicochemical, and dynamic properties of relatively large molecular systems at atomistic resolution. The all‐atom Newtonian MD simulation method is the most widely employed computational modeling technique to explore the structure, dynamics, and intermolecular interactions of complex systems comprising tens of thousands of atoms (Figure 2d–g).^[^ ^32^, ^34^ ^]^ Accurate prediction of molecular properties using classical MD is achieved by deriving or training the FF parameters using experimental data and/or QM calculations. The quality of FF parameterization is an essential consideration for reliable classical MD simulations, as highlighted by many FF comparison studies.^[^ ^35^, ^36^, ^37^, ^38^, ^39^ ^]^ Multiscale simulation approaches which combine both QM and MM methods (QM/MM) are applicable to systems where chemical reactions occur in solution or within the crowded molecular environment (Figure 2c).^[^ ^40^ ^]^ For larger multicomponent systems, classical MD simulation in coarse‐grained (CG) approximation is often employed, whereby a group of atoms is treated as a single interaction site (“bead”) thus reducing the number of particles treated (and the resolution of the model) but enabling access to longer biologically relevant timescales (Figure 2h). The latter is an important consideration when performing MD simulations of biosystems and is largely dependent on the method employed for sampling the highly rigged potential energy landscapes of multicomponent systems as determined by the FF.^[^ ^30^, ^41^, ^42^ ^]^ For spontaneously evolving time‐dependent MD simulations (often referred to as “brute force”), the efficient sampling of conformational space within reasonable timescales is challenging.^[^ ^43^, ^44^ ^]^ The rise in computer power and the development of enhanced sampling algorithms enabled the sampling of wider phase space for complex systems. Some commonly used enhanced sampling techniques, including umbrella sampling (US),^[^ ^45^ ^]^ replica exchange‐molecular dynamics (REMD,^[^ ^46^ ^]^ or REST^[^ ^47^ ^]^/REST2^[^ ^48^ ^]^) and metadynamics (MetaD),^[^ ^49^, ^50^ ^]^ have been recently reviewed for their utility in studying peptide self‐assembly in solution and in the presence of a nanosurface,^[^ ^30^ ^]^ and are applicable to the systems of interest herein.

In this perspective, we profile recent theoretical modeling simulations that highlight the potential of 2D graphene‐based NMs for biomedical applications. This complements the previous experimental^[^ ^2^, ^14^, ^15^, ^16^, ^19^, ^20^, ^23^, ^51^, ^52^, ^53^, ^54^, ^55^, ^56^ ^]^ and theoretical modeling^[^ ^4^, ^5^, ^11^, ^57^, ^58^, ^59^, ^60^, ^61^, ^62^, ^63^ ^]^ perspectives that covered a different range of 2D materials (i.e., molybdenum disulfide, tungsten disulfide, hexagonal boron nitride, black phosphorous, and MXene) or other carbon‐based NMs (i.e., nanotubes, fullerenes, and quantum dots). This review also complements and builds upon the previous theoretical modeling perspectives^[^ ^4^, ^5^, ^11^, ^57^, ^58^, ^59^, ^61^, ^62^, ^63^, ^64^, ^65^, ^66^, ^67^ ^]^ that have reviewed pioneering and notable computational modeling studies examining the interactions of 2D graphene‐based NMs with peptides/proteins,^[^ ^68^, ^69^, ^70^, ^71^, ^72^, ^73^, ^74^, ^75^, ^76^, ^77^, ^78^, ^79^, ^80^, ^81^, ^82^, ^83^, ^84^, ^85^, ^86^, ^87^, ^88^, ^89^, ^90^, ^91^, ^92^, ^93^, ^94^, ^95^, ^96^, ^97^, ^98^, ^99^, ^100^, ^101^, ^102^, ^103^, ^104^, ^105^, ^106^, ^107^, ^108^, ^109^, ^110^, ^111^ ^]^ nucleic acids,^[^ ^112^, ^113^, ^114^, ^115^, ^116^, ^117^, ^118^, ^119^, ^120^, ^121^, ^122^, ^123^, ^124^, ^125^, ^126^, ^127^, ^128^, ^129^, ^130^, ^131^, ^132^, ^133^, ^134^, ^135^, ^136^, ^137^, ^138^, ^139^, ^140^, ^141^, ^142^, ^143^, ^144^, ^145^, ^146^, ^147^, ^148^, ^149^, ^150^, ^151^, ^152^, ^153^, ^154^, ^155^, ^156^, ^157^, ^158^, ^159^, ^160^, ^161^, ^162^, ^163^, ^164^, ^165^, ^166^ ^]^ lipid membranes,^[^ ^69^, ^167^, ^168^, ^169^, ^170^, ^171^, ^172^, ^173^, ^174^, ^175^, ^176^, ^177^, ^178^, ^179^, ^180^, ^181^, ^182^, ^183^, ^184^, ^185^, ^186^ ^]^ and other biomedically relevant small molecules.^[^ ^187^, ^188^, ^189^, ^190^, ^191^, ^192^, ^193^, ^194^, ^195^, ^196^, ^197^, ^198^, ^199^, ^200^, ^201^, ^202^, ^203^, ^204^, ^205^ ^]^ Here, we focus on the most recent contributions of theoretical modeling to this fast‐developing field including graphene‐based surfaces and nanoparticles (graphene, GO, and rGO) but excluding nanotubes, fullerenes, and quantum dots that have been reviewed previously.^[^ ^59^, ^60^, ^61^, ^63^, ^67^, ^206^, ^207^, ^208^, ^209^, ^210^ ^]^ The interactions of 2D graphene‐based NMs with physiologically relevant biomolecular systems including peptides, proteins, nucleic acids, lipid membranes, and other biomedically important molecules are discussed in light of the computational advances and challenges as well as the promising applications of these NMs in biomedicine.

## 2 Solvent and Ionic Strength Effects on GNM Solubility

**Figure 3:**
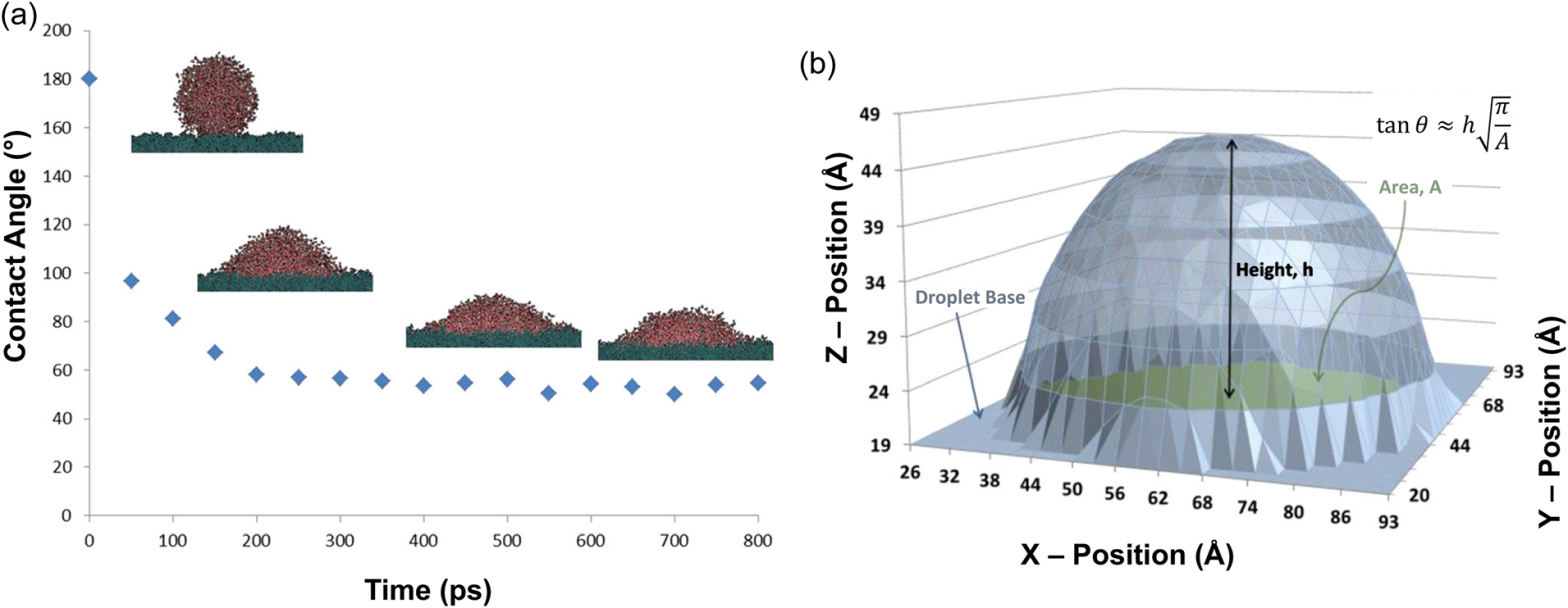
All‐atom MD simulations of spreading of a water droplet on graphene surface to determine water contact angle. a) A spherical water droplet was placed on graphene, and simulations were performed until the contact angle of the droplet stabilized. b) Three‐dimensional (3D) maps of the water droplet were constructed to determine nanoscale contact angle. Adapted with permission.^[^ ^211^ ^]^ Copyright 2014, American Chemical Society.

Understanding the interactions of 2D GNMs with aqueous solution affords insights into the behavior and solubility of these materials in biological environments. Graphene adhesion and wettability have attracted attention for biomedical as well as industrial applications and the modeling techniques have been developed to characterize nanoscale contact angle for graphene.^[^ ^211^, ^212^, ^213^, ^214^, ^215^, ^216^, ^217^ ^]^ A series of simulations of spreading of a water droplet on graphite were performed and contact angle measurements were extracted from three‐dimensional (3D) maps of the droplets (**Figure** **3**).^[^ ^211^ ^]^ The nanoscale contact angle measurements were related to macroscopic contact angle measurements, showing good agreement with experimental values of contact angle.

**Figure 4:**
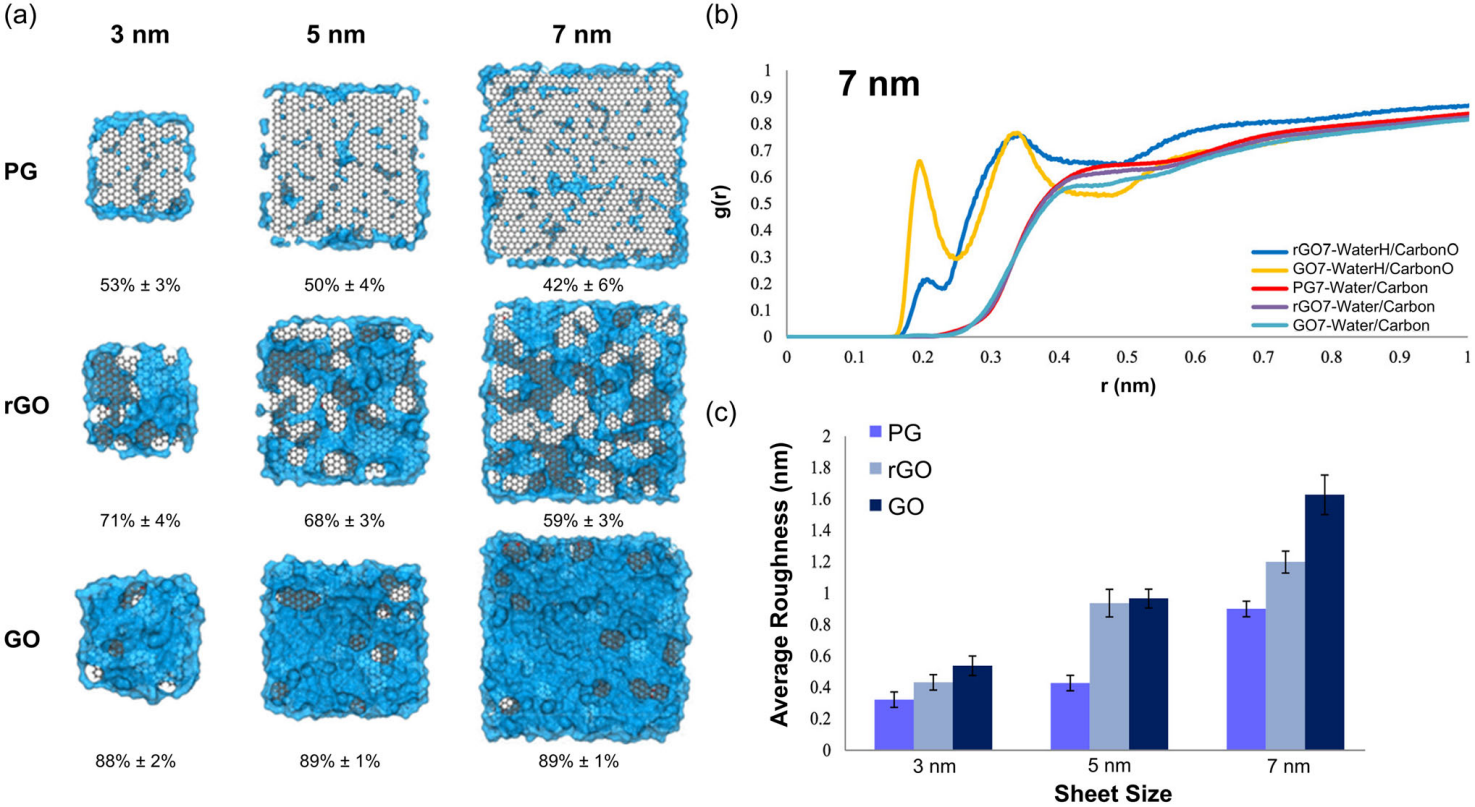
Exemplar snapshots from all‐atom MD simulation investigating the influence of graphene‐based NF size and oxidation degree on their structure, dynamics, and interactions in aqueous solution. a) Water coverage fraction (%) of PG, rGO, and GO NFs of different sizes: 3 × 3, 5 × 5, and 7 × 7 nm^2^. Water shown in blue, GNMs in gray color. b) NF–water radial distribution functions identifying hydration shells around the 7 × 7 nm^2^ NFs. c) Quantifying the atomic roughness of graphene‐based NFs with respect to NF size and oxidation. Adapted with permission,^[^ ^218^ ^]^ https://pubs.acs.org/doi/10.1021/acsomega.8b00866, Copyright 2018, American Chemical Society. Any further permissions related to this material should be directed to the American Chemical Society.

The ability of 2D GNMs to remain suspended in solution and avoid aggregation is important for their stability and low toxicity in biological fluids.^[^ ^22^ ^]^ Several explicit solvent all‐atom MD simulation studies elucidated the structuring of water around 2D GNMs. Despite application of different FFs and structural models, these studies showed that increasing the surface oxygen concentration on 2D GNMs leads to an increased water structuring around the particle, driven by the formation of hydrogen bonds between the oxygen‐containing groups and water.^[^ ^218^, ^219^, ^220^ ^]^ Wei et al.^[^ ^219^ ^]^ demonstrated that the contact angle of water absorbed onto graphene‐based surfaces decreases with increasing concentration of the surface oxygen groups. The surface roughness and oxygen patterning of GO also influenced the wetting and spreading behavior of water molecules over the surface. To understand the role of surface size and degree of oxidation on the behavior of 2D GNMs in aqueous medium, Peng et al. modeled nine graphene‐based NFs of different size (3–7 nm) and degree of oxidation (pristine, rGO, and GO) in all‐atom detail in aqueous solution (**Figure** **4**a).^[^ ^218^ ^]^ The authors found that larger NFs with a higher degree of oxidation enhanced the surface roughness and curvature of the particle (Figure 4c). Analysis of water structuring showed that the higher surface oxygen concentration areas induced greater water affinity within the first hydration layer, leading to the formation of distinct hydrophilic and hydrophobic patches on GO (Figure 4a,b) that could potentially be exploited to modulate protein adsorption. Such insights into the interactions of the GNMs with water can inform the substrate design for targeted interactions with biomolecules in the biomedical devices (in vitro) as well as in the biological environments (in vivo).

Experimental studies indicated that the toxicity of GO to living organisms in the aquatic environment is related to its aggregation propensity.^[^ ^221^ ^]^ Therefore, the solution behavior of 2D GNMs, such as their tendency for aggregation, is an important consideration for the design of soluble and safe NMs. All‐atom MD simulations were used to examine the influence of surface functionalization on the mechanism of GO aggregation.^[^ ^222^, ^223^, ^224^ ^]^ These studies showed that increasing the hydrophilicity of GNMs through the introduction of oxygen‐containing functional groups reduces their aggregation propensity. To understand the influence of surface oxidation on the aqueous dispersibility of GO, Bansal et al.^[^ ^224^ ^]^ modeled the interactions of water with two parallel GO sheets varying in the type and concentration of oxygen‐containing functional groups (hydroxyl, epoxy, and carboxyl). By calculating the potential of mean force (PMF) between the two GO sheets, strong repulsive forces between the GO sheets were found to prevent the aggregation of GO in water, with this repulsion increasing with higher concentrations of epoxy and hydroxyl groups. In contrast, when GO was functionalized at the edges with carboxyl groups, attractive forces between the basal bare graphene reduced GO dispersibility in water. The authors concluded that the hydroxyl‐functionalized GO sheets were most stable in water, as indicated by the PMF values indicative of increased interparticle repulsion and increased formation of intra‐ and interlayer water‐mediated hydrogen bonds indicating improved solubility.

Another all‐atom MD study found GO functionalized with hydroxyl groups aggregated faster than carboxyl‐functionalized GO due to the hydroxyl groups promoting greater number of GO–GO hydrogen bond,^[^ ^223^ ^]^ however, the binding energy between the GO flakes was not calculated. Another study used atomistic MD simulations to investigate the impact of GO oxidation pattern (i.e., peripherally or fully oxidized flakes) on its aggregation behavior in water.^[^ ^225^ ^]^ The simulations revealed that adjusting the GO oxidation pattern can alter the structural features of the formed aggregates, including the interlayer distance between sheets and the size and clustering of the formed GO aggregates. Specifically, the separation distance between sheets was greater, and the cluster sizes of GO sheets in the aggregate were larger for the fully oxidized flakes compared to the edge‐functionalized flakes. More recently, Sun et al.^[^ ^226^ ^]^ investigated the influence of the heterogeneous distribution of oxygen‐containing functional groups on GO aggregation in solution. The spontaneous assembly of one small GO NF with homogenous oxidation and one larger GO NF with distinct oxidized and nonoxidized panels was investigated using atomistic MD simulations. The heterogeneous distribution of oxygen‐containing functional groups resulted in different water structuring around the oxidized and unoxidized regions on GO, which subsequently led to different modes of aggregation at the different NF regions. Specifically, it was found that the aggregation of GO can occur via two mechanisms: either by point‐falling‐line‐squeezing‐plane (PFLSP) process, or by confined bridge water molecules. The PFLSP process involved one GO flake falling onto another, leading to the squeezing out of water molecules from between the two flakes and the formation of a strongly bound aggregate, while the confined bridge water molecules resulted in a larger interlayer distance between the GO sheets due to the presence of the water layer leading to a weaker interaction energy.

Considerable theoretical modeling effort has focused on modeling the aggregation of 2D GNMs in solution under different conditions (i.e., pH, presence of ions, metals, and surfactants). The pH‐dependent aggregation of GO was explored in a comparative experimental and atomistic MD simulation study^[^ ^227^ ^]^ examining the effects of pH on the colloidal stability and surface activity of GO in water. The experiments showed that in low pH conditions, distinct large‐scale GO aggregates were formed in solution, while in high pH conditions, these aggregates were not visible. Atomistic MD simulations uncovered the mechanisms behind the pH‐dependent aggregation of GO observed experimentally. The simulations showed that the protonated carboxyl groups at low pH reduced the hydrophilicity of GO, resulting in the formation of suspended GO aggregates in water, while increasing the pH reduced the formation of GO aggregates due to electrostatic repulsion between the deprotonated carboxyl groups. Another study^[^ ^222^ ^]^ examined the aggregation of two graphene and GO sheets in different solutions using all‐atom MD and density functional theory (DFT) simulations. Aggregate structures with similar interlayer distances and sandwich‐like configurations were observed by both simulation methods, indicating agreement between the atomistic MD simulations and DFT calculations. The MD simulations revealed that factors such as pH conditions, presence of metal ions, and the NM model size influenced the GNMs aggregation behavior. The presence of metal cations accelerated aggregation through surface adsorption, with smaller GNMs aggregating more quickly than larger ones, and high pH conditions reducing the tendency for GO aggregation due to electrostatic repulsion between deprotonated carboxyl and hydroxyl functional groups, a finding consistent with earlier studies.^[^ ^227^ ^]^ Furthermore, in a combined computational (MD and DFT) and experimental study,^[^ ^228^ ^]^ lower pH was also observed to accelerate GO aggregation, driven by *π*–*π* stacking interactions and the weakening of water‐mediated steric hindrance. Using experiments and DFT calculations, Xiang et al.^[^ ^229^ ^]^ showed that the dispersibility of graphene in aqueous environments can be increased with the use of rGO as a surfactant. The DFT calculations explained the experimental findings by showing the electrostatic repulsion between rGO and graphene to promote the increased dispersibility of graphene in aqueous solution, with surface oxygen concentration of rGO being an important parameter in maintaining the repulsion.

During the GO synthesis and purification, highly oxidized polyaromatic carboxylated fragments or oxidation debris (OD) can adhere to the GO surface and modify its structure and properties in solution as well as induce cytotoxic effects.^[^ ^230^, ^231^, ^232^, ^233^ ^]^ As such, Tang et al.^[^ ^234^ ^]^ recently used a combination of experiments and all‐atom MD simulations to understand the influence of OD on GO aggregation at high pH conditions. In the simulations at pH 14, electrostatic repulsion was found to induce the stripping of OD from the deprotonated hydroxyl‐rich basal plane of GO, while OD remained absorbed at the GO edges. The stripped OD then underwent aggregation in solution, followed by their readsorption near the edges of GO to form GO–OD aggregates. The inclusion of OD on the GO surface was found to accelerate GO aggregation, and the GO aggregation process was driven by the bridging effect of ODs, direct and water‐mediated hydrogen bonds, and GO–OD *π*–*π* stacking interactions. The bridging effect of ODs observed in the MD simulations was confirmed by atomic force microscopy (AFM).

### 2.1 Modeling Considerations and Challenges

Accurate atomistic representation of GO requires the inclusion of surface defects which are often created during the manufacturing and modification processes of graphene.^[^ ^235^, ^236^ ^]^ The influence of surface defects on the wettability of graphene and GO was explored by all‐atom MD simulations.^[^ ^237^, ^238^ ^]^ These simulations revealed that vacancy defects in GO reduce its wettability and increase the water contact angle,^[^ ^238^ ^]^ which is consistent with similar research on defective graphene.^[^ ^237^ ^]^ In a combined MD and DFT study, it was shown that the structural deformations and electronic properties of GO depend on the type and concentration of oxygen‐containing functional groups.^[^ ^239^ ^]^ This study also proposed that the common Lerf–Klinowski model needs to be revised to include 1,2‐ether groups on the GO basal plane. Moreover, theoretical models of GO often depict the oxygen‐containing functional groups to be randomly distributed on the basal plane. However, recent ab initio MD (AIMD) simulations showed that the random distribution of oxygen‐containing functional groups is energetically unfavorable.^[^ ^240^ ^]^ This study demonstrated that semiordered GO models with distinct clusters of oxygen‐containing functional groups were more stable in water than models with randomly ordered oxygen‐containing groups. Other approaches to generate more experimentally agreeable nonhomogeneous GO structures for atomistic simulations involve the use of machine learning (ML) algorithms.^[^ ^241^ ^]^

In addition to optimizing the model structure of 2D GNMs, computational research effort has focused on developing accurate FF parameters for studying their behavior in aqueous environment. For example, Pinto et al.^[^ ^242^ ^]^ took the GAFF2 FF bonded GO parameters and added new nonbonded parameters from linear‐scaling DFT calculations through an atoms‐in‐molecules (AIM) partitioning scheme, producing the GAFF2‐AIM FF. The new FF parameters reproduced the solvent structure reported in AIMD simulations better than commonly used other FFs. Specifically, the new FF induced a weaker solvent structure near the GO surface, which in turn influenced the binding mechanism of amino acids (AAs) to GO in further simulations. In another study, Neto et al.^[^ ^220^ ^]^ used atomistic MD simulations to compare approaches to modeling the atomic charges of GO by investigating the interactions between water and seven GO models differing in surface oxygen concentration (10–70%). The two models involved a simple charge approach by treating the *sp* ^2^ carbons as Lennard–Jones (LJ) uncharged sites and, a QM‐derived CHELPG charge model. While the structure of the hydration layer around GO was observed to be unaffected by the model employed, the simplified (no charge) model overestimated the GO–water interaction energy compared to the more accurate CHELPG charge model, also affecting the number of hydrogen bonds formed within the modeled systems. To enhance the empirical description of the graphene–water interaction, Feng et al.^[^ ^243^ ^]^ used DFT methods to develop a registry‐dependent interlayer potential (ILP) and applied the new FF parameters in MD simulations to evaluate the ILP efficacy. The water contact angles calculated from the MD simulations were within the experimental range, indicating the ILP capacity to describe the hydrophilicity of graphene. Recently, an extensive review of common FFs used to study the wetting of graphene and graphite revealed that most of them do not accurately reproduce experimentally measured contact angles.^[^ ^244^ ^]^ In light of this, a series of classical MD simulations were performed to determine the optimal carbon–oxygen dispersion energy that accurately reproduces the experimentally measured contact angles for water on graphene (80°) and graphite (60°). This work highlights the importance of FF quality for reliability of classical approaches in studying graphene–water interactions.

Other studies focused on including polarization effects when modeling the interactions between graphene and water. In an early study, Ho and Striolo^[^ ^245^ ^]^ conducted a series of all‐atom MD simulations on systems comprised of a thin layer of rigid single point charge extended (SPC/E) water or polarizable simple 4‐site water with Drude polarizability (SWM4_DP) on either nonpolarizable or Drude‐particle polarizable graphene. The simulation results showed that both water models produced similar structural properties of water on neutral nonpolarizable graphene, with slightly different results for the water dynamics. In contrast, the number of interfacial SWM4_DP water molecules increased on the neutral polarizable graphene model. When the simulations were performed using charged graphene, the effects of graphene polarization on water structure and dynamics were negligible due to electrostatic forces being more dominant than the polarization effects. In all cases, the polarizability of water and graphene was not observed to impact the hydrogen bond network. In another study,^[^ ^246^ ^]^ MD simulations were carried out using ab initio‐derived FF parameters to describe the polarization of graphene during wetting by water. Using a self‐consistent model of graphene polarization resulted in a remarkably higher and more experimentally agreeable water contact angle compared to implicitly modeled polarization using an LJ potential. Such differences in water contact angle were explained by variances in the water interfacial energy. More recently, Escalona et al.^[^ ^247^ ^]^ used charge‐on‐spring (COS) models to explore the induced polarizability of the graphene/water interface under different electric field conditions. The polarizable systems included COS polarizable graphene models and compatible polarizable GROMOS FF water models. The results for the polarizable systems were compared with nonpolarizable graphene/water models. In bulk water and no external electric field applied, no difference in the water–graphene interactions was observed between the polarizable and nonpolarizable systems. However, when an electric field was applied, differences in the structure and dynamics of water on graphene were evident with inclusion of polarizability. The water contact angle was found to differ depending on the water model, with the polarizable water models inducing a higher water contact angle than the nonpolarizable water model. Further, introduction of the polarizable graphene model resulted in a reduced water contact angle compared to nonpolarizable graphene, highlighting the importance of including polarizability for graphene‐water contact angle calculations. It was concluded that, given the steep computational costs of the COS polarizable models, the use of these complex models is justified when increased electrostatic responsiveness is important.

**Figure 5:**
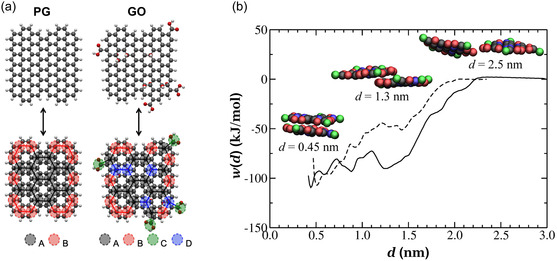
CG MD simulations investigating the aggregation of 2D GNMs. a) Atomistic reference models for PG and GO (top) and mapping method used to produce the CG representation (bottom). b) Potential of mean force for GO aggregation obtained from US simulations using the atomistic (dashed line) and CG (solid line) FFs. Adapted under terms of the CC‐BY 4.0 license.^[^ ^250^ ^]^ Copyright 2020, The Authors. Published by IOP Publishing Ltd.

CG models of 2D GNMs contributed to improved understanding of the mechanical properties of graphene and GO.^[^ ^248^, ^249^ ^]^ Williams and Lísal^[^ ^250^ ^]^ developed flexible CG solution‐based models for graphene and GO sheets that are compatible with the polarizable Martini water model^[^ ^251^ ^]^ (**Figure** **5**a). The radial distribution of water around the GNMs and the sheet–sheet aggregation free energies were accurately reproduced by the CG model (Figure 5b). Simulations of the sheets sliding over another revealed important mechanisms of graphene and GO aggregation/exfoliation in solution. Entropy was identified as a factor that prevents GO aggregation, while facilitating its dispersion in water. Notably, a 20‐fold increase in computational efficiency was achieved using the CG model to study GNM aggregation compared to atomistic representation, highlighting the important role of CG simulations for understanding the GO assembly in solution.

Assessing the structure and behavior of 2D GNMs for biomedical applications also requires knowledge of their interactions with ionic species present in physiological environments. Theoretical modeling techniques were applied to study the interfacial structuring and dynamics of ions in solution on graphene‐based surfaces. Computational studies showed that ions form a dense and persistent interfacial layer over graphene,^[^ ^242^, ^252^, ^253^, ^254^, ^255^, ^256^ ^]^ where the ion–*π* interactions formed via ion‐induced polarization effects significantly contribute to the observed behavior.^[^ ^252^, ^254^, ^257^ ^]^ However, the modeled adsorption and structuring of Cl^−^ and Na^+^ ions on GO vary depending on the FF model chosen.^[^ ^242^, ^256^ ^]^ Specifically, the FF model can influence the density of ions around the surface as well as the preferential ion adsorption sites to different types of oxygen‐containing functional groups. Accurate prediction of the interactions between ions and GNMs, therefore, requires inclusion of polarizability for both the surface and solution models. Williams et al.^[^ ^252^ ^]^ proposed a new method to parametrize classical FFs using DFT calculations to incorporate polarizability of graphene and ions using a simple pairwise potential. The new model successfully reproduced the polarizability effects consistent with the first principles (DFT) calculations, while avoiding the use of computationally expensive polarizable potentials. Specifically, the inclusion of cation–π interactions into the new model resulted in greater structuring of ions over the graphene surface, indicating the importance of including electrolyte−*π* interactions to accurately describe the graphene‐ions interfaces. Another methodology enabling improved modeling of the ion‐induced polarization of graphene included a QM/MD coupling approach.^[^ ^258^ ^]^ This method involved the electronic structure calculations of the graphene surface using the density functional tight binding (DFT‐TB) calculations coupled with the classically represented (all‐atom MD) electrolyte solution. The approach was shown to be computationally cost‐effective in modeling uncharged and charged graphene in a NaCl solution, revealing the preferential adsorption of cations over anions on the charged surfaces. Recently, using atomistic MD simulations,^[^ ^255^ ^]^ the Drude polarizable FF was employed to investigate the electrolyte–graphene interactions and the influence of the ion size on their surface adsorption mechanism. While smaller ions were found to directly adsorb onto graphene, the larger ions formed a structured ion shell around the graphene surface. Notably, the Drude parameters captured anion–graphene interactions that are often underestimated using nonpolarizable FFs, demonstrating the role polarization plays in the adsorption of Cl^−^ ions onto graphene.

## 3 Small Molecule Functionalization of GNMs for Biomedical Applications

**Figure 6:**
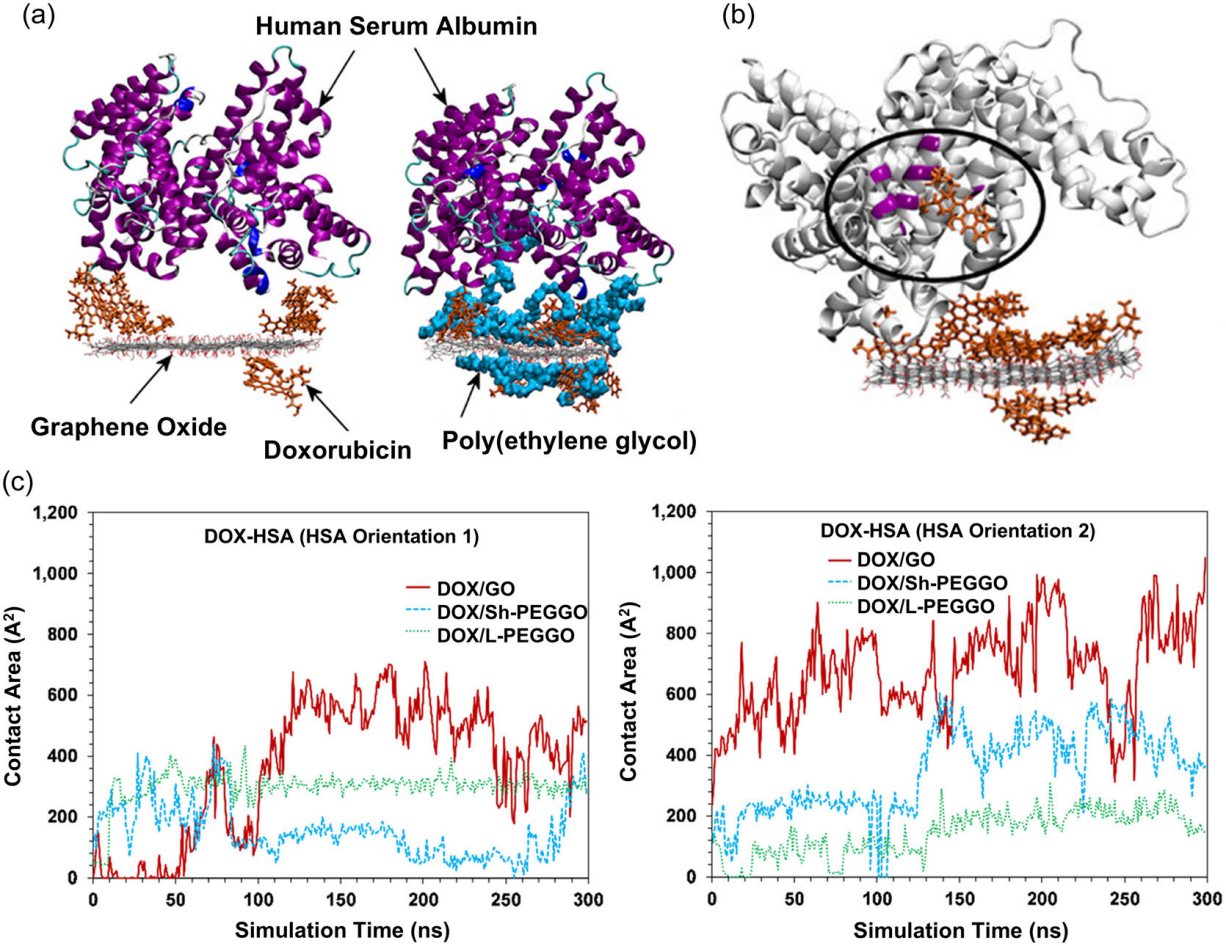
All‐atom MD simulation examining the interactions between GO, doxorubicin (DOX), and HSA for the design of a drug–GO nanocarrier system. a) Molecular snapshots of initial drug–GO systems containing GO loaded with DOX and HSA (left), and with the addition of PEG (right). b) Molecular snapshot showing the release of DOX from the DOX‐loaded GO system, driven by the DOX–HSA interaction. c) Evolution of contact area between DOX and HSA during the simulations, where a high contact area indicates the release of DOX. Adapted with permission.^[^ ^263^ ^]^ Copyright 2020, American Chemical Society.

A promising biomedical application of 2D GNMs is their use as nanocarriers for targeted drug delivery.^[^ ^259^, ^260^ ^]^ Theoretical modeling techniques were applied to study the interactions between drug candidates and 2D GNMs, providing insights into the nanocarrier stability and function. For instance, DFT and MD simulations were employed to investigate applications of 2D GNMs for carrying and releasing small drug molecules for treatment of cancer^[^ ^187^, ^188^, ^189^, ^190^, ^199^, ^200^, ^201^, ^202^, ^203^ ^]^ and viral infections.^[^ ^191^ ^]^ Using all‐atom MD simulations and the Molecular Mechanics Poisson‐Boltzmann Surface Area (MM‐PBSA) method for calculating the binding free energy, Hashemzadeh and Raissi^[^ ^261^ ^]^ found coloading of anticancer drugs doxorubicin (DOX) and paclitaxel (PTX), resulted in stronger drug adsorption on graphene than on GO, consistent with an earlier all‐atom MD simulation study.^[^ ^262^ ^]^ It was also shown that under low pH conditions both drug molecules were released from the nanocarrier surfaces due to the electrostatic repulsion. However, in the presence of a lipid membrane, the GO nanocarrier was capable of spontaneously penetrating the membrane, while the graphene system was not. This suggested that drug–GO systems may be more effective at drug release in the cellular environment. Another all‐atom MD simulation study showed that DOX stability and retention on human serum albumin (HSA)‐loaded GO can be enhanced by grafting polyethylene glycol (PEG) chains onto the GO particle to produce PEGylated GO (PEGGO) nanocarriers (**Figure** **6**a).^[^ ^263^ ^]^ It was observed that GO is more prone to release DOX prematurely compared to PEGGO (Figure 6b,c), and that longer PEG chains promoted greater drug retention on GO surfaces (Figure 6c). More recently, using DFT calculations, PEGGO was identified as an ideal carrier for the antiviral drug 4′‐Fluorouridine in acidic tumor environments, owing to its ability to enhance complex stability and promote controlled release kinetics.^[^ ^264^ ^]^

Another all‐atom MD simulation study showed that functionalizing GO with chitosan (CS) to produce a GO–CS nanocarrier system may improve GO's PTX adsorption capacity by promoting PTX–nanocarrier hydrogen bonding.^[^ ^262^ ^]^ Further, atomistic simulations were applied to examine the influence of temperature (25, 35, 45, and 55 °C) during the delivery of the anticancer drug cyclophosphamide (CP) on CS/graphene nanocomposites.^[^ ^265^ ^]^ The calculated CP diffusion coefficient was higher as the temperature increased. Further, the CP drug delivery systems containing CS and nitrogen (N)‐doped graphene nanosheets attained a lower CP diffusion coefficient at the highest temperature compared to systems containing pure graphene and phosphorus (P)‐doped graphene. This indicated that the CS/N‐doped graphene nanocomposite may be more efficient for controlled drug diffusion out of all the tested nanocarrier surfaces. The influence of temperature on the adsorption and desorption of benzene on graphene was also explored in another recent study using AFM experiments and MD simulations.^[^ ^266^ ^]^ The adsorption capacity between graphene and benzene was found to decrease linearly with increasing temperature, suggesting that the adsorption and desorption process of benzene‐containing drug molecules on graphene can be controlled by tuning the temperature environment.

In a combined DFT and classical MD simulation study,^[^ ^267^ ^]^ Arumugam et al. found that the adsorption energy of the anticancer drugs lapachol and β–lapachone on graphene increased by up to 70% when surface defects were introduced and by about 50% when the surface was chemically doped with silicon. Furthermore, the drug‐loaded defective and doped graphene sheets were more stable than PG. In another study,^[^ ^268^ ^]^ spontaneous MD and well‐tempered metadynamics (MetaD) simulations were employed to investigate the influence of Poly (L‐histidine) (PLH) polymer attachment to graphene on the adsorption capacity of the anticancer drug gemcitabine (GMC). The simulation findings indicated that stronger GMC adsorption can be achieved when graphene is covalently functionalized with PLH, and that PLH may aid drug release under low pH conditions. Other reported applications of the theoretical molecular modeling to study drug/GNM nanocarrier systems include the anticancer drugs curcumin,^[^ ^269^ ^]^ cytarabine and clofarabine,^[^ ^270^ ^]^ the antidiabetic drug miglitol,^[^ ^271^ ^]^ and the antiviral drug heparin.^[^ ^272^ ^]^

Molecular simulations were also applied to explore potential exploitation of 2D GNMs as sensors for disease biomarkers,^[^ ^192^, ^193^, ^194^, ^195^, ^196^ ^]^ and as nanoenzymes.^[^ ^204^, ^205^ ^]^ For example, the first principles (quantum chemical) calculations were applied to investigate the peroxidase mimicking activity of graphene and seven GO models varying in oxygen‐containing functional groups (epoxy, hydroxyl, ether, endoperoxide, carbonyl, carboxyl, and ester).^[^ ^273^ ^]^ Specifically, DFT calculations were used to study the catalytic mechanism involving the oxidation of 3,3,5,5‐tetramethylbenzidine (TMB) by hydrogen peroxide (H_2_O_2_). Out of all the studied GO models, the DFT results indicated the carbonyl group as the active catalytic center, and identified activation of the C=O bond as the key catalytic step.

## 4 GNMs Interactions with Peptide and Proteins: From Cytotoxicity Effects to Functional Biomaterials

Theoretical modeling of the interactions of AAs, peptides, and complete proteins with the GNMs was shown to be helpful not only for optimizing their potential applications in nanotechnology and biomedicine but also for a better understanding of potential cytotoxic effects of GNMs, as discussed in the following sections.

### 4.1 Amino Acid–GNM Interactions: Basic Models and FFs

**Figure 7:**
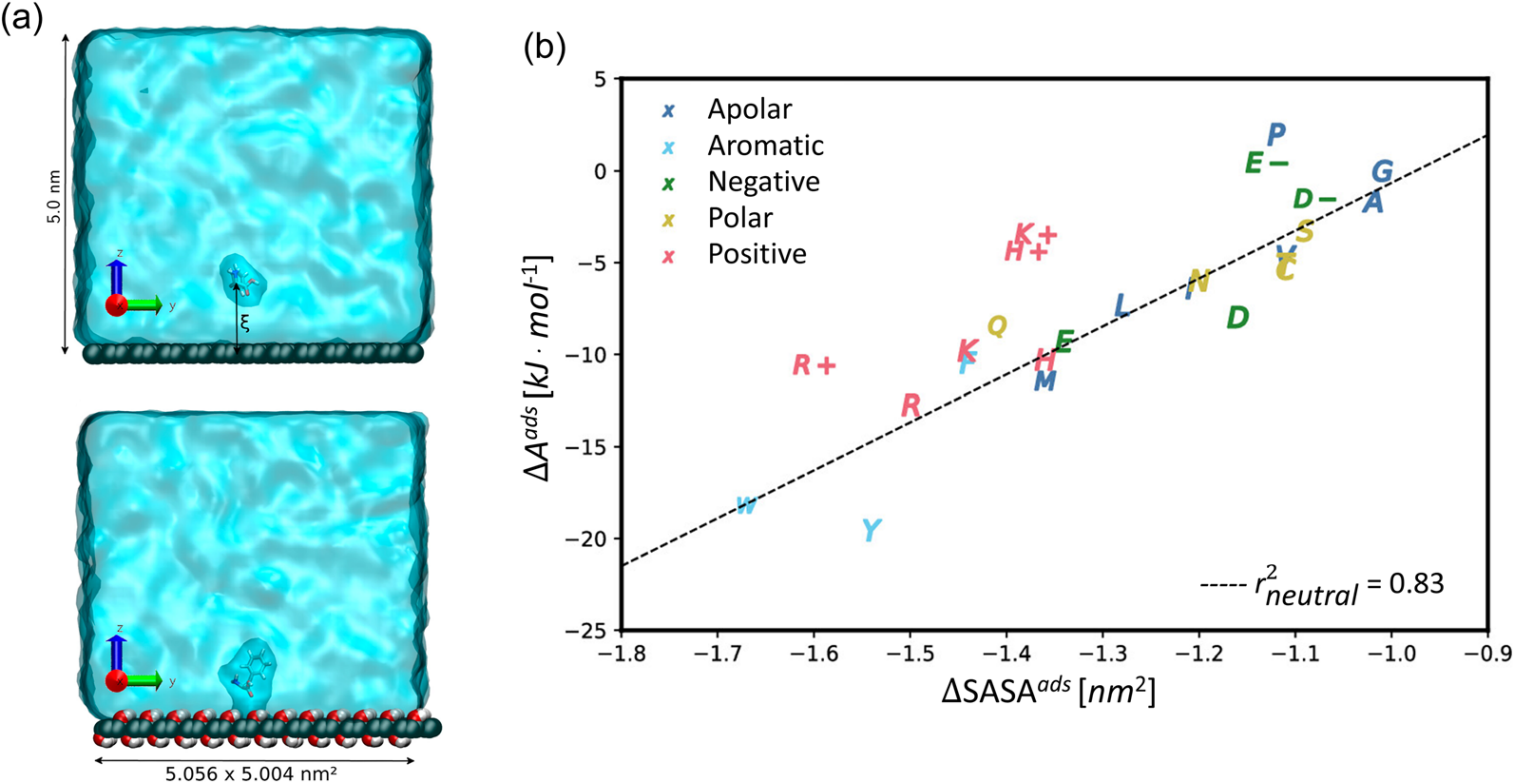
The influence of AA size on graphene interactions. a) An AA interacting with PG (top) and GO (bottom) in solution. b) Reduced adsorption‐free energies (Δ*A* ^ads^), with Δ*A* ^ads^ of Gly set to zero, as a function of reduced solvent‐accessible surface area ΔSASA^ads^ for all AAs over PG, revealing a linear correlation between the two variables for the neutral AAs. Adapted with permission.^[^ ^276^ ^]^ Copyright 2023, American Chemical Society.

Modeling the adsorption of AAs on 2D GNMs provides insights into the binding affinity of individual AA sidechains which can help optimize the peptide/protein binding on the NMs. For example, in the in vacuo MD simulations,^[^ ^274^ ^]^ aromatic AAs were observed to exhibit the strongest total interaction energy with graphene due to *π*–*π* stacking contributions, while Arg with its guanidinium group was found to be the strongest binder among the aliphatic, polar, and charged AAs. To account for the solvent effects and conformational variability, Hughes and Walsh^[^ ^77^ ^]^ used MetaD simulations to calculate the binding free energies of all twenty naturally occurring AAs to graphene in solution. Arg, Tyr, Trp, Gln, and Gly AAs were found to be most strongly bound, while Ile was the weakest binder. Considering these binding free energies, it was suggested that AAs with large, planar groups such as phenol, indole, and guanidinium, as well as those with compact side chains like Gly, promote strong adsorption. This was explained by their sidechain geometries helping to avoid contact with the first hydration layer, while AAs with bulky sidechains like Ile were weaker binders due to greater contact with the first water layer. In another study, generating the binding free energy data of twenty proteinogenic AAs on graphene from umbrella samplings (US) MD simulations, Hirano and Kameda^[^ ^275^ ^]^ created an “aromaphilicity” index which defined the affinity of each AA sidechain for the aromatic carbon surface. The index revealed aromatic AAs and Arg had the largest aromaphilicity, in good agreement with experimental data and the previous study.^[^ ^77^ ^]^ The authors suggested that this index could be used to predict the stability of proteins on aromatic surfaces.^[^ ^275^ ^]^ Recently, it was shown using all‐atom MD simulations and free energy calculations that the binding free energy of uncharged AAs on graphene was correlated with the size of the sidechain (**Figure** **7**).^[^ ^276^ ^]^ The results of AA binding affinity to graphene correlated well with prior studies^[^ ^77^, ^275^ ^]^ despite different FF and MD sampling methods. Furthermore, a prominent driving force in AA adsorption involved the removal of water from the graphene surface, leading to strong AA–graphene interaction.^[^ ^276^ ^]^ In another study, Xue et al.^[^ ^277^ ^]^ used atomistic MD with US simulations to explore the entropic and enthalpic contributions to the adsorption of six AAs (Ala, Asn, Asp, Phe, Thr, and Lys) on graphene. It was found that adsorption of AAs of small molecular size (Ala, Thr, and Asp) was mostly driven by the enthalpy change, while for larger AAs (Phe, Asn, and Lys) it was mainly driven by the change of entropy. The entropy gain observed for larger AAs was contributed to their size promoting the removal of more water molecules from the hydration layer during surface adsorption. Interestingly, this entropy‐driven adsorption was not due to structural changes for the larger AAs, whereas for smaller AAs, more conformational changes and less water removal were observed during AA adsorption.

Simulation studies showed that the addition of oxygen‐containing functional groups to the PG surface affects AA binding. Using both DFT or MD methods, it was found that inclusion of oxygen‐containing functional groups on the graphene‐based surface decreased the parallel orientation of the aromatic AAs^[^ ^140^, ^274^ ^]^ due to reduced *π*–*π* stacking interactions, while the binding of charged and polar AAs was favored. In another study, all‐atom MD simulations and free energy calculations showed that the inclusion of oxygen‐containing functional groups led to less favorable sidechain adsorption and a reduction in the size correlation observed for the graphene‐containing systems shown in Figure 7.^[^ ^276^ ^]^ These findings demonstrated the enhanced biocompatibility of GO compared to graphene. Recently, MetaD simulations were employed to study the adsorption of AAs and AA chain analogs,^[^ ^278^ ^]^ where the backbone was removed and the C_α_ carbon replaced by a hydrogen,^[^ ^279^ ^]^ on different 2D GNMs, including graphene, GO, and rGO. Aromatic and cyclic AA sidechains were found to preferentially bind to pure graphene, while polar and charged molecules exhibited lower binding affinity.^[^ ^278^ ^]^ The presence of oxygen‐containing functional groups in GO was observed to influence the adsorption mechanism of the AA analogs, with charged molecules binding most strongly on the functionalized graphene‐based surfaces via electrostatic interactions. However, a higher surface oxygen concentration was observed to promote weaker biomolecule binding due to the water structuring around the functional groups. These studies demonstrated that the adsorption behavior of biomolecules can be controlled by tuning the surface functionalization of NMs as well as the chemical properties of the biomolecules.

The AAs can self‐assemble on graphene to form nanopatterned surfaces which holds promise for developing improved biomaterials for biomedical applications.^[^ ^30^ ^]^ In two separate studies,^[^ ^280^, ^281^ ^]^ Awuah and Walsh used all‐atom MD simulations to investigate the spontaneous self‐assembly of AAs on graphene, revealing insights into the influence of AA type and termination on the formed adlayer pattern morphology. In the first study,^[^ ^281^ ^]^ in vacuo simulated annealing MD simulations were performed of systems containing neutral, zwitterionic, and neutral–zwitterionic mixtures of Trp and Met on graphene. The results showed the formation of a dimer row pattern over graphene by the zwitterionic AA systems, termed the canonical dimer row, while the neutral adlayers did not form any ordered patterns. In a follow‐up study,^[^ ^280^ ^]^ the authors investigated the self‐assembly of neutral, zwitterionic, and neutral–zwitterionic mixtures of Ala and His adsorbed on graphene in vacuo. In agreement with the earlier study,^[^ ^281^ ^]^ zwitterionic‐containing AA systems were necessary for formation of the ordered AAs adlayers.^[^ ^280^ ^]^ However, in contrast to the previous study, where only the canonical dimer row pattern was formed,^[^ ^281^ ^]^ the MD simulations of Ala and His on graphene showed the co‐existence of several pattern motifs.^[^ ^280^ ^]^ Based on the finding of both studies,^[^ ^280^, ^281^ ^]^ the authors proposed that the pattern of assembled AAs on graphene can be predicted by their sidechain characteristics, including compactness and hydrogen bonding capacity. Specifically, AAs with sidechains that are not compact, do not support hydrogen bonding, and bind strongly with graphene, are more likely to form the canonical dimer row patterns.

#### 4.1.1 Modeling Considerations and Challenges

The depiction of AA (and subsequently peptide/protein) adsorption onto GNMs is often influenced by the FF model used. Pinto et al.^[^ ^242^ ^]^ developed new nonbonded parameters from QM calculations to more accurately model GO in aqueous environments and applied the new FF parameters to estimate the adsorption energies of AAs onto GO. The new FF parameters produced AA binding free energies comparable to experiments and captured the size‐dependent binding strength of AAs over GO. However, since the new FF parameters enabled a weaker solvent structuring near the GO surface, some AAs, including Lys, Glu, Asp, were found to have preference for the bulk solvent rather than the surface. This finding emphasizes how the interplay of interactions between GO, water, and the AAs in classical MD simulations is highly dependent on the FF parameterization. To assess the effects of FFs on the protein–graphene interactions, Dasetty et al.^[^ ^282^ ^]^ employed all‐atom MD with US simulations to compare the structural preferences of AAs on graphene using common nonpolarizable FF models, such as Amberff99SB‐ILDN, CHARMM36, OPLS‐AA/M, and Amber03w. The AAs were found to favorably interact with graphene regardless of the FF and AA type. PMFs constructed from the US simulations showed qualitative similarities between the FFs and experimental data available. However, the simulated adsorbed AA structures were FF‐dependent.

Another important consideration in the development of FFs for studying protein–graphene interactions is the inclusion of polarizability which can influence the binding process. The polarizable AMOEBAPRO^[^ ^283^ ^]^ FF has been extended for studying the interactions between proteins and GNMs like CNTs.^[^ ^284^ ^]^ However, despite its high quality, the AMOEBA FF is considered computationally demanding which limits its application for modeling protein adsorption.^[^ ^77^, ^285^, ^286^ ^]^ To address this FF challenge, Hughes et al.^[^ ^287^ ^]^ developed the polarizable GRAPPA FF for graphene and CNTs that is compatible with the CHARMM FF. GRAPPA utilizes a rigid‐rod model for describing polarizability, whereby a single dummy atom with a small charge is attached to each real carbon atom. Following this study,^[^ ^287^ ^]^ the authors applied the GRAPPA FF with enhanced sampling simulations to investigate AA–graphene and peptide–graphene interactions, elucidating the link between peptide sequence and conformation on the experimentally determined graphene‐binding capacity of peptide sequences.^[^ ^77^ ^]^ Interestingly, another group found the experimental agreement of the polarizable GRAPPA FF for modeling the adsorption of alanine dipeptide on graphene was lower than that of the standard CHARMM FF.^[^ ^288^ ^]^ These discrepancies highlight the challenges in developing transferable FF models that capture the structure, dynamics, and polarizability of biomolecular–NM interactions in various environments.^[^ ^289^ ^]^

### 4.2 Effects of GNMs on Protein Secondary Structure and Self‐Assembly

All‐atom MD simulations showed that proteins can quickly adsorb onto PG due to Van der Waals (VdWs), hydrophobic, and *π*–*π* stacking interactions with graphene.^[^ ^70^, ^71^ ^]^ The strong binding between proteins and graphene can disrupt the secondary structure of proteins, highlighting potential toxicity of the graphene‐based materials. For instance, using MD simulations, the capacity of graphene to unfold α‐helices was documented for protein GB1,^[^ ^74^ ^]^ a domain of a cell surface protein which binds to immunoglobulin G (IgG), and the villin headpiece protein (HP35),^[^ ^75^, ^76^, ^290^, ^291^ ^]^ a common protein model used in MD simulations. Protein unfolding was linked to the formation of dominant hydrophobic and vdWs protein–graphene interactions which caused the complete loss of the hydrophobic core important for maintaining the tertiary structure of HP35.^[^ ^290^ ^]^ Other MD simulation studies showed that the planar surface of graphene contributes to the helical breakdown by enabling strong binding of residue sidechains through the extension of AAs over its large (predominantly flat) surface area.^[^ ^68^, ^76^, ^81^, ^292^ ^]^ In contrast, all‐atom MD and US simulations revealed wrinkled (curved) graphene accelerated protein unfolding of HP35 compared to planar graphene.^[^ ^291^ ^]^ This was explained by the ability of HP35 to better conform to the curved surface and result in higher binding affinity compared to the planar areas. Further, the wrinkled region promoted restricted transverse movement of bound aromatic residues, while other protein regions were free to move, causing protein denaturation, suggesting wrinkled graphene may be more disruptive to the HP35 structure than planar graphene. Other potential cytotoxic effects of graphene, as documented by atomistic MD simulations, include graphene's ability to block the catalytic chamber of an adsorbed proteasome protein,^[^ ^293^ ^]^ as well as interrupt hydrophobic protein–protein dimer interactions that are essential for important biological functions.^[^ ^93^ ^]^

Modeling the interactions between peptides/proteins and 2D GNMs presents an opportunity to understand how the surface properties of GNMs can be tuned to mediate the structure and behavior of proteins. Computational modeling studies found that the presence of oxygen‐containing functional groups on GO promotes formation of a stable hydration layer compared to graphene, leading to more conserved native protein conformations during protein adsorption.^[^ ^73^, ^294^ ^]^ Moreover, GO's oxygen‐rich surface introduces opportunities for hydrogen bonding and electrostatic interactions, resulting in various mechanisms of protein adsorption compared to PG.^[^ ^294^ ^]^ Recently, atomistic MD simulations revealed the secondary structure of skin keratin protein was better conserved on GO compared to graphene, indicating biocompatibility of GO.^[^ ^295^ ^]^ In another study,^[^ ^296^ ^]^ all‐atom MD simulations applied to understand the effects of GO functionalization on apolipoprotein‐cIII (apo‐c3) adsorption provided insights into protein corona formation and the role of protein–NM interactions in reducing the toxicity of the material. Two types of functionalized GO were considered, including standard GO and azide‐ and alkyne‐double functionalized graphene oxide (C2GO). Analysis of the secondary structure evolution during the protein adsorption revealed that GO induced considerable denaturation of the apo‐c3 structure, driven by the electrostatic interactions. Adsorption of apo‐c3 on GO led to formation of three individual β‐turns that could serve as templates for protein–protein interactions and promote the protein corona formation which could hinder the targetability of the NM in the biological environment. In contrast, C2GO was found to be better at maintaining the tertiary protein structure in the adsorbed state. This was explained by the weaker protein–surface interactions due to the azide functional groups sterically hindering the charged protein residues. It was concluded that adsorption of apo‐c3 onto C2GO could conserve the native structure and in turn lipid‐binding capacity of the protein.

In addition to the surface chemistry of GNMs, the intrinsic peptide properties, such as AA sequence, length, and secondary structure, were found to dictate the peptide adsorption process. Using experiments as well as REMD and adaptive biasing force (ABF) simulations, Kuang et al.^[^ ^297^ ^]^ examined the impact of AA substitution, peptide sequence, and solution pH on the graphene edge‐binding capacity of a peptide model (native sequence: EPLQLKM).^[^ ^78^, ^298^ ^]^ AFM experiments showed the native peptide preferentially binds to graphene's edges and the replacement of N‐terminal Glu with Gly as well as shuffling the native AA sequence resulted in different peptide binding behavior on the graphene/graphite surface. PMF calculations using the ABF algorithm supported the experimental findings and showed the lowest energy state occurred when the peptide was bound to the graphene edge, driven by the interaction between the carboxylate group of Glu and the zigzag graphene edges. The PMF calculations demonstrated that N‐terminal protonation and Gly substitution reduce the edge‐binding capacity of the native peptide due to reduced electrostatic interactions. Furthermore, the PMF results showed the edge‐binding capacity of the native peptide was lower when the peptide sequence was randomly shuffled, demonstrating that the native peptide sequence with N‐terminal deprotonated Glu exhibited the highest binding affinity for the graphene edges.

Substantial evidence suggests that adsorption of peptides/proteins onto graphene and GO can be harnessed for the development of next‐generation biomedical devices and therapies. For instance, in a combined experimental and atomistic MD simulation study,^[^ ^299^ ^]^ the application of GO in cancer therapy was explored. The MD simulations showed that a GO particle was able to insert into the interstrand gap of an actin tetramer, resulting in the disruption of the actin filament, which was also confirmed experimentally. Since the actin cytoskeleton plays an important role in regulating cell migration, these findings suggested that GO could retard cancer cell migration by acting as an actin disruptive binding agent. In a recent study,^[^ ^300^ ^]^ atomistic MD simulations of graphene (G) and functionalized graphene (G—COOH, G—NH_2_, G—COOH—NH_2_, G—OH, and G—O) interacting with the calcitonin gene‐related peptide receptor revealed the most stable receptor binders followed the order G—OH > G—COOH > G. These GNMs were found to disrupt the receptor protein structure, indicating these nanosheets can be promising candidates for the treatment of migraines by inhibiting the binding of the calcitonin gene‐related peptide hormone to its receptor and thus lessening the pain symptoms of a migraine.

**Figure 8:**
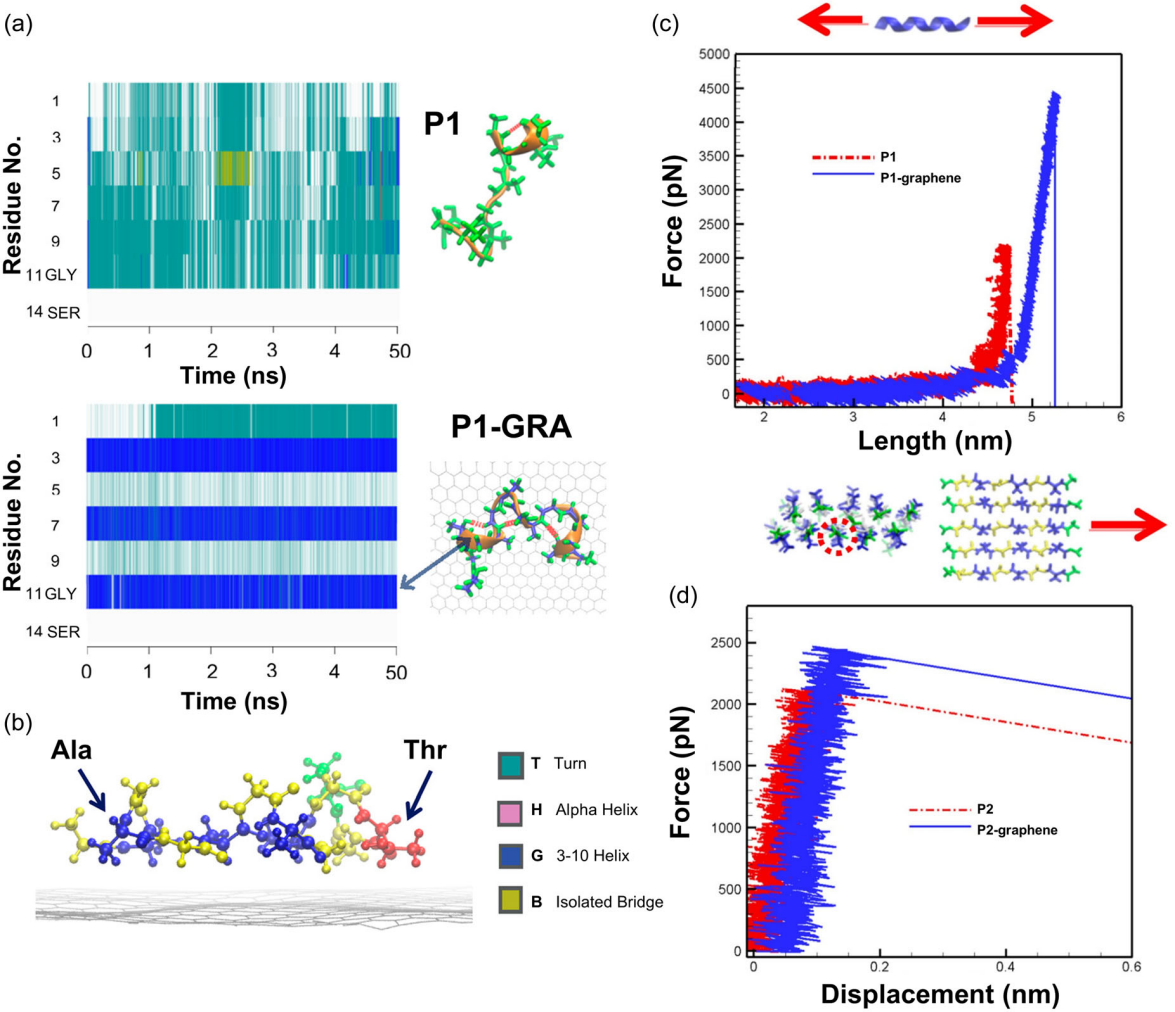
All‐atom MD simulation examining the interactions between silk fibroin peptides and graphene. a) Evolution of silk fibroin peptide (P1) secondary structure without (top) and with (bottom) graphene. b) Molecular snapshot showing the interaction between P1 and graphene. AAs are colored based on their names: Ala is blue, Gly is yellow, Thr is red, and Ser is green. c) Results of tensile test showing the pulling force as a function of P1 end‐to‐end distance. d) Results of SMD simulations showing the pull‐out force as a function of peptide displacement. Adapted with permission.^[^ ^301^ ^]^ Copyright 2015, American Chemical Society.

Atomistic MD simulations demonstrated graphene can enhance the mechanical properties of silk fibroin,^[^ ^301^ ^]^ a suitable material for bone or ligament tissue engineering.^[^ ^302^, ^303^ ^]^ This was observed through time‐evolved MD simulations, which showed that graphene promotes the formation of helical structures in silk fibroin domains, whereas turn and coil structures were observed in the absence of graphene (**Figure** **8**a). The graphene‐enhanced mechanical strength of silk fibroin was further shown using theoretical tensile tests and steered MD (SMD) simulations. In the tensile tests, a force was applied to pull a single peptide strand (Figure 8c), while in the SMD simulations, a force was applied to extract a peptide strand from the protein (Figure 8d). A higher rupture force and peptide resilience were found with the presence of graphene (Figure 8c,d), highlighting that graphene can be used to design bioinspired materials with improved mechanical properties. Recently, experiments and MD simulations in both all‐atom and CG representation were applied to study the growth of silk fibroin nanofibril on graphene.^[^ ^304^ ^]^ All‐atom MD simulations were used to investigate the initial stages of silk fibroin self‐assembly on graphene. The results showed when silk fibroin adsorbed on graphene it formed β‐sheet structures parallel to the graphene surface. The assembly process was also faster on graphene than in solution, where temperature played an important role in accelerating the surface‐mediated assembly process, as confirmed by experiments. In another study,^[^ ^305^ ^]^ all‐atom MD simulations were used to explore the binding of silk light and heavy chains to PG. The study found silk light chain adsorption to graphene was driven by hydrophobic interactions and resulted in structural maintenance of silk light chain. Moreover, this interaction increased the hydrophilicity of graphene due to the exposure of the hydrophilic surface in the adsorbed silk light chain protein. These simulations findings indicated the biocompatibility of graphene was improved via silk light chain functionalization, which was further confirmed by experimental contact angle measurements.

Recent research efforts focused on investigating the binding strength of materials‐binding peptides and peptide analogs to graphene. Using DFT calculations, Guo et al.^[^ ^86^ ^]^ demonstrated that including surface defects and oxygen‐containing functional groups on the graphene surface can promote stronger binding of the RGD tripeptide compared to PG. Further, replica‐exchange with solute tempering (REST) simulations were employed to study the graphene binding affinity of experimentally determined 2D NM binding peptides and their analogs: BP1 (LLADTTHHRPWT), BP7 (VDAQSKSYTLHD), and P1 (HSSYWYAFNNKT).^[^ ^306^ ^]^ The study found BP1 and PB7 had similar binding affinities to graphene, while P1 was most strongly bound. While aromatic residues were found to contribute greatly to the binding of BP1 and P1 to graphene, the contact strength of Trp differed depending on its position in the peptide sequence. In a series of experiments and REST simulations,^[^ ^307^, ^308^ ^]^ Walsh and colleagues explored how the conjugation of fatty acid chains to the P1 peptide affects the peptide's adsorption on graphene. These studies showed that the adsorbed P1 structure and binding affinity to graphene is influenced by the fatty acid attachment point, at either the P1 N‐terminus or C‐terminus.

Atomistic MD simulations were used to examine the self‐assembly of peptides on graphene‐based surfaces which can help design novel biocompatible materials. For example, β‐sheet‐containing peptides on graphene‐based surfaces, revealing the orientation of the assembled β‐sheets, were dictated by graphene's symmetry.^[^ ^309^ ^]^ No et al.^[^ ^310^ ^]^ reported a nature‐inspired approach to design 2D self‐assembled peptides on PG. This was accomplished by first performing structural statistical analyses, including the interchain distance and intraresidue distance, of naturally occurring β‐sheet structures to construct a nature‐inspired β‐sheet‐forming peptide backbone. Atomistic MD simulations were then employed using the constructed backbone with different peptide sequences to determine the optimal peptide sequence for the formation of a stable 2D peptide assembly on graphene. The MD simulations revealed that among the studied peptide sequences (K_4_F_4_, K_4_Y_4_, K_4_W_4_, and K_4_V_4_) the most optimal sequence for 2D assembly on graphene was the antiparallel stacking of K_4_F_4_ peptides. Another atomistic MD simulation study examined the self‐assembly of diphenylalanine on graphene in aqueous conditions.^[^ ^311^ ^]^ The peptide assembly process was found to occur via the formation of a stable interfacial layer of diphenylalanine on graphene, followed by the formation of another self‐assembled diphenylalanine layer on top. The interfacial layer of diphenylalanine on graphene was stabilized by *π*–*π* stacking interactions between the phenyl rings and graphene surface. In lieu of experimentally resolved structural data of the self‐assembled peptide structure at the aqueous graphene interface, Hughes and Walsh^[^ ^312^ ^]^ employed REST simulations of the GrBP5 peptide (IMVTESSDYSSY) and its mutant sequences adsorbed on graphene in solution. The adsorption of a single GrBP5 peptide on graphene was driven by the aromatic‐rich C‐terminal region. Simulations considering multiple peptide chains revealed the formation of unordered nano‐patterns with patches of exposed bare graphene, whereby the aggregation process was driven by interchain hydrogen bonding and electrostatic interactions. These simulation findings were in contrast to the ordered peptide assemblies observed experimentally on graphite, possibly due to those experiments involving a drying process while the simulations investigated peptide assembly in solution. In another study,^[^ ^313^ ^]^ Awuah and Walsh used simulated annealing MD simulations to investigate the self‐assembly of neutral, zwitterionic, and neutral–zwitterionic mixtures of either WA or AW dipeptides adsorbed onto graphene in vacuo. Regardless of the AA termination, all dipeptide systems exhibited spontaneous formation of dipeptide patterns over graphene. This finding was different to previous studies which showed that neutral AAs did not form ordered patterns over graphene compared to the zwitterionic‐containing AA systems.^[^ ^280^, ^281^ ^]^ Further, the WA dipeptide was found to have a greater propensity to form ordered patterns than the AW peptide, indicating the influence of the AA sequence on the peptide self‐assembly process. The simulation findings suggested the ability of dipeptides to form self‐assembled patterns over graphene is increased when the peptide sequence contains an AA with an extended sidechain with a low capacity to support hydrogen binding at the N‐terminus, while the C‐terminus includes a compact sidechain that supports no hydrogen bonding.

#### 4.2.1 Modeling Considerations and Challenges

While the above examples highlight the usefulness of MD simulations to complement and explain experimental observations by providing detailed atomistic information into the protein adsorption process on graphene‐based surfaces, the accurate description of these complex bio–nano systems with many degrees of freedom requires substantial statistical sampling of the potential energy landscape. Studies investigating the sampling performance of spontaneous MD simulations and enhanced sampling MD methods to investigate protein–surface interactions have highlighted the challenges of using spontaneous MD simulations to adequately sample conformational changes across high energy barriers within accessible timescales.^[^ ^314^, ^315^, ^316^, ^317^ ^]^ Spontaneous MD and accelerated MD (aMD) simulations examining the interactions between the bone morphogenetic protein 2 (BMP‐2) and graphite showed that the biasing aMD technique was important in observing the complete unfolding and spreading of BMP‐2 on graphite.^[^ ^315^ ^]^ In another study, Ou et al.^[^ ^316^ ^]^ showed that the experimentally observed α‐helix to β‐sheet transition in an α‐helical peptide (DELERRIRELEARIK) adsorbed onto graphene was not featured within the timeframe of the 200 ns spontaneous all‐atom MD simulations, even at higher 310–330 K temperatures. More recently, we compared the adsorption behavior of inherently disordered protein, human islet amyloid polypeptide (hIAPP), on graphene‐based NFs varying in physical dimension and surface oxygen concentration using both spontaneous MD and bias‐exchange MetaD (BE‐MetaD) simulations.^[^ ^317^ ^]^ We found that BE‐MetaD was able to sample adsorbed amylin states exhibiting fewer helical structures, greater protein–NF contact area, and a higher number of α‐helix to β‐sheet transitions compared to spontaneous MD. These findings demonstrated the importance of applying enhanced sampling techniques such as BE‐MetaD in modeling the structure and binding of inherently disordered proteins to surfaces and nanoparticles.

Below, we discuss theoretical modeling case studies for specific peptides/proteins interacting with 2D GNMs including blood proteins, amyloidogenic proteins, virus‐related proteins, and enzymes.

#### 4.2.2 Protein Interactions with GNMs: Case Studies

##### Blood Proteins

Atomistic MD simulations showed that adsorption of blood proteins, including bovine fibrinogen, immunoglobulin (Ig), transferrin (Tf), and bovine serum albumin (BSA), onto graphene is dominated by *π*–*π* stacking and hydrophobic interactions as well as basic residue contacts.^[^ ^72^, ^84^, ^85^ ^]^ A combined experimental and all‐atom MD simulation study^[^ ^318^ ^]^ investigated the adsorption of typical serum proteins, including HSA, Ig E (IgE), and apolipoprotein E (ApoE), on graphene and hydroxylated graphene. The simulations showed that the contact area between HSA and IgE with the graphene‐based surfaces was inversely correlated to the amount of hydroxyl groups present on the surface, while ApoE had similar contact area with all graphene‐based surfaces, suggesting the ApoE adsorption process was not influenced by the surface hydrophilicity. These simulation findings agreed with the experimental results showing greater HSA and IgE adsorption due to the addition of hydroxyl groups on graphene, while ApoE was found to interact similarly with all three GNM surfaces. Compared to proteins in solution, the adsorbed serum proteins exhibited a decrease in ordered secondary structure elements (helix or β‐sheet) due to the reconfiguration of proteins during surface adsorption. However, for adsorbed IgE and HSA, the reduction of the ordered secondary structure was most prominent on the pristine surfaces, indicating that the PG surface promoted the conformational restructure of the protein enabling a greater protein–surface contact area. This suggested that the introduction of hydroxyl groups on the graphene‐based surface can reduce the extent of protein denaturation, and thus better preserve the biological function of these proteins. In contrast, similar secondary structures were observed during the adsorption of ApoE on the graphene‐based surfaces, matching the results for the contact area, and suggesting that the biological function of ApoE can be preserved on all graphene‐based surfaces.

Using all‐atom MD simulations, Jo et al.^[^ ^319^ ^]^ suggested graphene may be cytotoxic to a blood‐coagulation protein (tissue factor/FVIIa) due to its capacity to penetrate through the lipid membrane which causes instability of the membrane‐bound protein. In contrast, all‐atom MD simulations showed that the toxicity of GO can be reduced by using surface coatings of BSA which reduces GO's cell membrane penetration capacity.^[^ ^69^ ^]^

Investigating the interactions between serum proteins and graphene can facilitate the design of graphene‐based biomedical devices. For instance, the binding of graphene with HSA and glycated HSA (GHSA), a diabetes biomarker, was recently investigated using all‐atom MD simulation and the MM‐PBSA method,^[^ ^320^ ^]^ to aid the design of a graphene‐based aptasensor for detecting GHSA. The protein adsorption onto graphene induced helical losses, and these structural changes were most prominent for GHSA. In addition to the secondary structure changes, graphene destroyed the function of albumin by inducing the release of glucose at the drug sites which meant the protein's ligand‐binding affinity was lost. The study concluded that using graphene as an aptasensor may require coverage of bare graphene to prevent GHSA–graphene interactions which would reduce the sensor's performance by interfering with the protein's structure and function. Another all‐atom MD simulation study^[^ ^263^ ^]^ studied the interactions between GO–DOX nanocarriers and HSA. The adsorption of HSA to PEGylated GO–DOX nanocarrier induced greater drug stability and retention than when the surface of GO was not PEGylated. These results indicate that GO PEGylation may be important in preventing the premature release of DOX during HSA adsorption.

##### Amyloidogenic Peptides and Proteins

The spontaneous misfolding and fibrillation of biological proteins induce amyloidosis, a pathological condition characterized by the abnormal buildup of cytotoxic amyloid protein fibrils in organs and tissues that disrupt their functions.^[^ ^321^ ^]^ The process of amyloidosis is linked to the onset and progression of many degenerative diseases including but not limited to, Alzheimer's, Parkinson's, type II diabetes, and atherosclerosis.^[^ ^322^, ^323^, ^324^, ^325^ ^]^ A promising biomedical application of 2D GNMs includes their use as therapeutic agents capable of modulating the protein (mis)folding/aggregation process, as demonstrated by various experimental studies.^[^ ^83^, ^326^, ^327^, ^328^, ^329^, ^330^, ^331^ ^]^ In vitro experiments showed GO delays the fibrillation of amyloid‐beta (Aβ), hIAPP, and HSA in solution,^[^ ^326^, ^327^, ^328^, ^331^ ^]^ and induces the clearance of mature Aβ fibrils.^[^ ^83^ ^]^ In vivo experiments found GO administration reduced Aβ accumulation in mice and improved Aβ‐induced cognitive impairment.^[^ ^329^, ^330^ ^]^ Theoretical modeling has been employed to explore the interactions between different amyloid proteins and GNMs, offering insights into the potential use of GNMs as fibril inhibitors. However, the precise mechanism of amyloid inhibition and the optimal design of the NMs capable of modulating protein aggregation remain unclear.^[^ ^332^, ^333^ ^]^

The last decade has focused on theoretical investigation of graphene as antiamyloid nanotherapies. Computational modeling studies showed graphene to exhibit a strong binding with different amyloidogenic peptides dominated by π–π stacking interactions.^[^ ^82^, ^334^ ^]^ Using all‐atom MD simulations, the adsorption of human islet amyloid polypeptide fragment 22–28 (hIAPP_22–28_) on graphene was observed to prevent β‐sheet formation and peptide self‐assembly by decreasing the hydrophobic peptide–peptide interactions, suggesting potential fibril‐inhibiting properties of graphene.^[^ ^82^, ^335^ ^]^ Similarly, a DFT study also showed that graphene nanosheets interact strongly with amyloid β‐sheets and this favorable interaction is stronger than the peptide–peptide binding within the β‐sheet.^[^ ^336^ ^]^ Another all‐atom MD simulation study revealed that graphene competes with the membrane‐binding capacity of hIAPP_22–28_ peptides and fibrils, and is even capable of extracting the membrane‐adsorbed biomolecules.^[^ ^335^ ^]^ The adsorption of the hIAPP fibril on graphene was driven by the receding of water (dewetting) at the fibril–graphene interface and the formation of *π*–*π* interactions, indicating the fibril‐inhibiting mechanism of graphene may be associated with the material acting as a clearing agent by removing membrane‐bound hIAPP_22–28_ and preventing membrane damage.^[^ ^337^, ^338^ ^]^ More recently, a study combined atomistic MD simulations and experiments to reveal the inhibitory effects of graphene on the aggregation of the prion protein segment 127–147 (Pr127–147).^[^ ^334^ ^]^ Consistent with other MD studies,^[^ ^68^, ^81^, ^82^ ^]^ the large carbon surface area of graphene was observed to induce strong peptide–graphene interactions which reduced interpeptide contacts and inhibited β‐sheet formation.^[^ ^334^ ^]^ These simulation findings provided mechanistic insights into the inhibition of prion protein aggregation by graphene, aiding in the interpretation of the thioflavin T fluorescence assay results that demonstrated graphene's inhibitory effects. The shape of graphene has also been observed to dictate its peptide adsorption capacity, where planar graphene surface promoted stronger protein binding compared to curved sheets.^[^ ^81^, ^82^ ^]^

**Figure 9:**
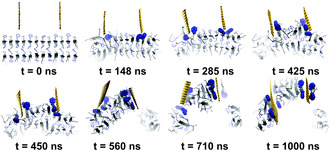
Molecular snapshots at different time intervals showing graphene penetrating and cutting a preformed amyloid‐beta (Aβ) fibril composed of twenty‐four Aβ peptide segment 16–21. Adapted with permission.^[^ ^83^ ^]^ Copyright 2015, The Royal Society of Chemistry.

The potential role of graphene in modulating amyloid disease is also associated with its ability to penetrate and cut mature fibrils.^[^ ^83^, ^339^ ^]^ MD and US simulations showed that graphene sheets can penetrate an Aβ fibril containing twenty‐four Aβ peptide 16–21 segments (Aβ_16–21_) (**Figure** **9**).^[^ ^83^ ^]^ The strong peptide–graphene interactions, driven by *π*–*π* stacking and hydrophobic interactions, resulted in the extraction of peptides from the fibril. The modeling findings were supported by AFM images and thioflavin fluorescence assays, which showed the capacity of GO to inhibit monomer aggregation and clear mature fibrils. The fibril‐destructive capacity of graphene was later also observed by Zhang et al.^[^ ^339^ ^]^ through all‐atom MD simulations examining the interactions between graphene and a fibril composed of the Aβ peptide 17–42 segment (Aβ_17–42_). The adsorption mechanism of the Aβ_17–42_ fibril onto graphene involved mostly VdWs interactions, as well as solvent‐mediated effects.

Conversely, other theoretical modeling studies demonstrated that graphene may indeed function as a fibril promoter, potentially inducing toxic effects by accelerating amyloid build‐up in organs. For example, a combination of the electronic structure calculation, classical MD, and US simulations were used to investigate the interactions between graphene and the apolipoprotein C‐II (apoC‐II) peptide fragment 60–70 (apoC‐II_60–70_).^[^ ^68^ ^]^ The adsorption of apoC‐II_60–70_ on graphene led to extended fibril‐forming peptide conformations, suggesting a possible fibril growth accelerating properties induced by graphene whereby the flat surface acts a template for peptide self‐assembly. The potential fibril‐promoting properties of graphene were further confirmed by another computational study using full‐length hIAPP as a protein model.^[^ ^317^ ^]^ The spontaneous MD and BE‐MetaD simulations indicated that large PG NFs (>5 nm) may accelerate hIAPP aggregation due to these surfaces facilitating unrestricted lateral mobility and promoting structural changes such as loss of native helical content and β‐sheet formation. The fibril‐promoting properties of graphene, including its capacity to induce protein misfolding and helical degradation, were also observed in a combined experimental and all‐atom MD simulations study of the Pr segment 125–228 (Pr_125–228_).^[^ ^340^ ^]^

The addition of oxygen‐containing functional groups on graphene enhances its dispersibility, stability, and biocompatibility in biological environments, as highlighted in the earlier section. As such, more recent research efforts have focused on the potential application of GO as amyloid modulators. Nedumpully‐Govindan et al.^[^ ^331^ ^]^ produced early theoretical insights into the antiamyloidogenic properties of GO using discrete MD simulations to examine the interactions between GO and full‐length hIAPP, in addition to their experimental techniques. The binding of GO with monomeric hIAPP led to a reduction in helical content and increase in coiled structures, in agreement with circular dichroism (CD) experiments. The adsorption process was driven by hydrogen bonding between polar residues and the oxygen‐containing functional groups as well as *π*–*π* stacking interactions between aromatic residues and the bare aromatic carbon rings on GO. From multipeptide simulations, the peptide–peptide contact number was found to decrease with the inclusion of GO, indicating there is a strong binding affinity of hIAPP for GO. At high peptide concentrations, the available contact area of GO was insufficient to induce permanent secondary structural changes in all peptides, with some helical content being restored in the peptides. The study concluded the amphiphilic nature of GO induced strong protein–surface interactions which may prevent the self‐association of hIAPP. Another study used REMD simulations to investigate the interactions between GO and full‐length Aβ dimers.^[^ ^341^ ^]^ β‐sheet formation in the Aβ dimers was reduced during the binding of Aβ with GO due to weakening of the interpeptide interactions. The driving forces of protein adsorption mostly included the formation of salt bridges, hydrogen bonds, and cation‐π interactions, in addition to the common protein–GO interactions such as *π*–*π* and hydrophobic interactions. In a recent study,^[^ ^342^ ^]^ all‐atom MD simulations were employed to investigate the interactions between GO and an Aβ fibril containing twelve monomer units. Aβ fibril adsorption onto GO caused fibril instability by interrupting Aβ–Aβ interactions important for the fibril structure. These findings, along with the previous fibril–graphene simulation studies discussed above,^[^ ^83^, ^339^ ^]^ suggest that 2D GNMs hold promise to disentangle preformed Aβ fibrils found in Alzheimer's disease.

Given that the fibril forming or inhibiting capacity of GNMs depends on their surface design features and protein sequence, it is necessary to evaluate each design factor and protein model using a systematic approach. To examine the impact of GO size on protein aggregation, Chen et al.^[^ ^343^ ^]^ used REMD simulations to explore the GO size effects on the fibrillation of two to four Aβ_33–42_ molecules. The presence of GO‐suppressed fibril‐prone β‐sheet formation reveals the fibril‐inhibiting properties of GO. However, the larger GO nanosheet (1.72 × 1.16 nm^2^) provided a larger contact surface area necessary for inhibiting β‐sheet formation and minimizing peptide–peptide interactions more effectively than the smaller GO nanosheets (1.13 × 1.13 nm^2^). These simulation findings agree with an earlier experimental study which found larger GO sizes delay Aβ_33–42_ aggregation compared to smaller GO NPs.^[^ ^327^ ^]^

In addition to the NF size, the oxygen concentration on the GO surface has been shown to influence its fibril‐modulating capacity. For instance, using atomistic MD simulations, Baweja et al.^[^ ^344^ ^]^ observed rGO with a carbon‐to‐oxygen (C:O) ratio of 10:1 more effectively inhibited the conformational transitions of Aβ_1–40_ than GO with a C:O ratio of 4:1. This study highlighted the importance of the distinct hydrophobic patches on the rGO surface in reducing the dynamics of peptide adsorption. These findings by Baweja et al. were further supported in a recent study examining the effect of 2D GNMs on hIAPP fibrillation.^[^ ^317^ ^]^ Using the enhanced sampling simulation method BE‐MetaD, the interactions between full‐length hIAPP and nine graphene‐based NFs varying in size (3, 5, and 7 nm) and C:O ratios (1:0, 10:1, 5:1) were explored. The simulation showed that the oxidized graphene‐based NFs form hydrogen bonds between the surface oxygen regions and hIAPP, leading to restricted protein mobility and a decreased ability of β‐sheet formation. This is in contrast with the pure graphene substrate which promoted considerable unrestricted lateral mobility and structural changes such as helical breakdown and β‐sheet formation. A lower GO surface oxygen concentration (C:O of 10:1) was observed to be more effective at reducing the conformational flexibility of hIAPP and preserving the native hIAPP structure compared to the GO surface with a high surface oxygen concentration (C:O of 5:1). These findings indicated that the oxygen concentration and surface patterning of graphene‐based NFs impacts the hIAPP binding. Specifically, the presence of large regions of unfunctionalized carbon and lower surface hydration of graphene‐based NFs with a high C:O ratio was found important for “locking” adsorbed hIAPP in place. In contrast, GO with a high surface oxygen concentration and surface hydration facilitated considerable conformational dynamics, leading to the extension of hIAPP over the GO NF surface. These atomistic insights suggested that the oxygen concentration and surface patterning of GO, in addition to the NF size, can be tailored to lead to modulate specific and robust protein binding.

**Figure 10:**
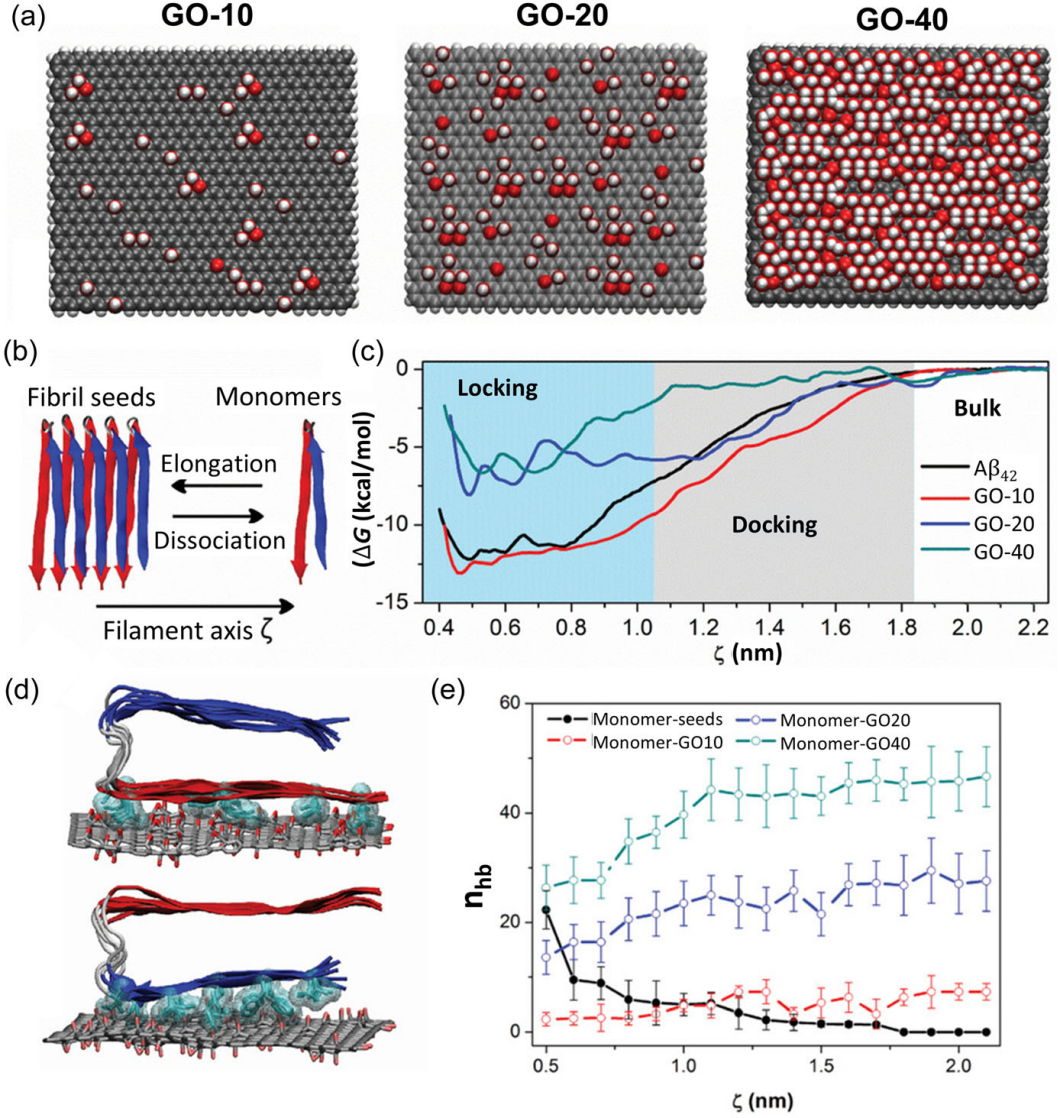
All‐atom MD simulation examining the interactions between an amyloid‐beta (Aβ) fibril and GO surfaces. a) Representative simulation models of GO varying in oxygen concentration (10, 20, and 40%). b) Schematic showing the process of Aβ fibril growth. c) Free energy profiles from US simulations showing the separation of peptide from the fibril seed along the filament axis. d) Representative structures from MD simulations of the Aβ fibril binding to GO with 10% oxygen concentration. e) Average number of monomer–seed and monomer–GO hydrogen bonds along the filament axis. Adapted with permission.^[^ ^345^ ^]^ Copyright 2021, John Wiley and Sons.

However, while there is evidence to suggest that GO surfaces with low oxygen concentration may be more effective at inhibiting amyloid fibril formation,^[^ ^317^, ^344^ ^]^ another atomistic MD simulation study showed the opposite trend for a different protein fibril–GO systems.^[^ ^345^ ^]^ In this study,^[^ ^345^ ^]^ experiments and a series of MD simulations were employed to examine the interactions between Aβ42 fibrils and GO surfaces varying in oxygen concentration (10, 20, and 40%) (**Figure** **10**a). Experimental data from thioflavin fluorescence experiments, calorimetric measurements, and transmission electron microscopy (TEM) observations, suggested that Aβ42 fibril growth was inhibited to greater extents by GO sheets with a higher oxidation degree. The MD simulations showed GO surfaces with oxygen concentration greater than 20% loosened the initial fibril structure via weakening the interpeptide interactions. The MD US simulations were also employed to investigate the diffusion of a single monomer to the GO‐bound Aβ_42_ fibril, elucidating the free energy profile for the fibril growth (Figure 10b,c). The fibril growth was observed to be less favorable on GO surfaces with higher oxidation levels due to the strong monomer–GO hydrogen bonds hindering the formation of crucial monomer–fibril hydrogen bonds that are necessary for fibril growth (Figure 10b,c,e). In contrast, when GO had a lower (10%) oxygen, fibril elongation was favored due the surface templating the docking and locking of incoming monomers to the Aβ_42_ fibril.

All‐atom MD simulations also showed a potential to tune the surface oxygen distribution on rGO and GO to optimize its Aβ fibril‐destructive capacity.^[^ ^346^ ^]^ Specifically, the nonuniform distribution of GO and rGO resulted in greater dissociation of Aβ_16−21_ oligomers compared to the uniformly distributed oxidized surfaces. This was attributed to formation of stronger monomer–surface interactions when the surface contained distinct hydrophobic and hydrophilic patches.

Further research has been conducted focusing on the influence of surface charge and chemical doping on the fibril‐inhibiting capacity of 2D GNMs. In particular, positively and negatively charged graphene surfaces were observed to inhibit α‐synuclein fibril formation to greater extent than neutral graphene.^[^ ^347^ ^]^ Moreover, MD simulations showed nitrogen (N)– and silicon (Si)‐doped graphene is more effective in preventing α‐synuclein aggregation than PG.^[^ ^347^, ^348^ ^]^

Overall, while the reviewed studies suggest 2D GNMs can be promising candidates for anti‐amyloid nanotherapies, they also highlight the challenges associated with formulating universal design criteria for NMs that are effective and specific toward different types of amyloidogenic proteins. Ideally, such agents need to possess the capacity to both breakdown the formed fibrils and protofibrils as well as, and possibly even more importantly, prevent the early stage fibril seed nucleation and growth of the toxic intermediate low molecular weight oligomers. As highlighted above, various factors such as the specific amyloidogenic peptide sequences and stages of the fibril formation can be explored theoretically together with the GNM design parameters such as the nanoparticle size, shape, and functionalization to enable a systematic and cost‐effective search of the efficient and safe amyloid inhibitors.

##### Viral Proteins

The COVID‐19 pandemic spurred research into the interactions between 2D GNMs and viral proteins relevant to SARS‐CoV‐2, exploring the potential of 2D GNMs for detecting and inhibiting of COVID‐19 infection.^[^ ^349^, ^350^ ^]^ Potential applications included the use of 2D GNMs in biosensors, personal protective equipment, antiviral coatings, and therapeutic strategies. Atomistic MD simulations were used to explore the interactions of graphene with the whole SARS‐CoV‐2 spike protein (S‐protein) monomer comprising the virus receptor binding domain (RBD).^[^ ^351^ ^]^ The 40 ns simulation of the 618 318 atom system showed that adsorption of the S‐protein onto graphene resulted in a more compact monomer shape while the RBD did not undergo significant secondary and tertiary structural changes during adsorption on graphene. Subtle changes to the RBD included unfolding of one α‐helix, while the other two α‐helices and the β‐sheet structures remained intact. Furthermore, the RBD residues involved in the angiotensin‐converting enzyme 2 (ACE2) interaction showed low binding affinity to graphene, resulting in their structural integrity during adsorption. In contrast, using atomistic MD simulations and the MM‐PBSA method, Du et al.^[^ ^352^ ^]^ reported that key residues involved in RBD–ACE2 binding on the RBD interface favorably interact with graphene, leading to significant alteration to the secondary structure of these RBD residues. Since SARS‐CoV‐2 infection is caused by the binding of the RBD to the ACE2, these RBD‐graphene interactions may influence the pathogenicity of SARS‐CoV‐2. The different RBD binding sites on graphene observed in both studies^[^ ^351^, ^352^ ^]^ may be related to the different protein subunits investigated, with the former study involving the entire S‐protein monomer,^[^ ^351^ ^]^ while the latter focused only on the RBD.^[^ ^352^ ^]^ Additionally, the initial RBD orientation toward graphene and the simulation timeframes (40 and 180 ns, respectively) varied significantly between the studies, which may explain the variation in results by the different degree of the conformational sampling. All‐atom MD simulations also revealed that graphene can insert in between the N‐terminal domain (NTD) and the RBD in their closed state, suggesting graphene may inhibit infection by blocking the RBD–ACE2 binding interface.^[^ ^87^ ^]^ Other MD studies predicted the interactions between the main protease (M^pro^) of SARS‐CoV‐2 and different 2D GNMs, revealing insights into the design of GNMs with increased inhibitory effects toward SARS‐CoV‐2.^[^ ^88^, ^89^ ^]^ These studies showed that defective graphene and functionalized P‐doped graphene may perform better at interfering with SARS‐CoV‐2 activity compared to PG. Aside from inhibiting infection, the interactions between 2D GNMs and SARS‐CoV‐2 S‐proteins were studied using theoretical modeling techniques to assess and advance the design of graphene‐based SARS‐CoV‐2 detection devices.^[^ ^90^ ^]^

Virus protein R (Vpr) is a HIV‐I accessory protein that contributes to virus replication and the pathogenesis of HIV‐1.^[^ ^353^ ^]^ Experiments showed that adsorption of the Vpr segment 13–33 (Vpr_13–33_) on GO may reduce Vpr cytotoxicity by breaking the α‐helix structure responsible for its function and by reducing the concentration of the virus in solution through surface adsorption.^[^ ^354^ ^]^ To gain atomistic insights into the Vpr–GO interactions, all‐atom MD simulations were employed to study the binding of Vpr_13–33_ monomer and dimer on GO.^[^ ^355^ ^]^ The favorable adsorption of Vpr_13–33_ on GO was driven by *π*–*π* stacking and electrostatic interactions. The adsorption process induced conformational changes including α‐helical breakdown and partial protein unfolding. PMFs generated from the US simulations identified differences in binding energy between Vpr_13–33_ dimers in solution and adsorbed on GO. The PMFs revealed the Vpr_13–33_ monomer binds stronger to GO compared to self in a dimer form in solution. The strong binding of the peptides on GO hindered the extent of peptide dimerization due to the peptides being “locked” in place on the oxygen‐rich surface.

Another study employed atomistic MD simulations to investigate the potential impact of graphene on the immune response to HIV.^[^ ^356^ ^]^ An essential aspect of the immune response during HIV‐1 infection involves the interactions between the antigenic peptide (KK10) and human leukocyte antigens (HLA), which, upon binding to T‐cell receptors, triggers a cellular response aimed at defending against HIV infection.^[^ ^357^ ^]^ The ability of graphene to interfere with the protein–protein interactions between the T‐cell receptor and the KK10–HLA complex was investigated using all‐atom MD simulations.^[^ ^356^ ^]^ The simulations demonstrated that graphene could disrupt the critical protein–protein interactions essential to this immune response system through hydrophobic and VdWs protein–graphene interactions causing the separation of the receptor from the KK10–HLA complex. Therefore, the use of graphene‐based biomedical devices may disrupt the immune response to HIV.

Besides HIV and SARS‐CoV‐2, other computational studies investigated a potential role of graphene in the spread of the Ebola virus.^[^ ^358^ ^]^ Atomistic MD simulations investigated the interactions between the Ebola matrix protein VP40 and graphene sheets. The simulations showed that graphene can insert and break apart the hydrophobic protein–protein interactions in the viral matrix filament, indicating the promising antiviral properties of the NM and the possible use of graphene as disinfectants to prevent viral spreading.

##### Enzymes

The interactions of NMs with enzymes were explored using theoretical modeling techniques, providing insights into the toxicological effects of these materials in the biological environment.^[^ ^67^ ^]^ Chen et al.^[^ ^359^ ^]^ used a combination of ML analysis and discontinuous MD simulations to study the interactions between lysozyme and graphene. Hydrophobic and *π*–*π* stacking interactions between the lysozyme residues and graphene were observed to drive adsorption and contribute to the collapse of the enzyme's tertiary structure. Recently, all‐atom MD simulations and the ABF method were used to study the adsorption and binding energy of hen egg white lysozyme (HEWL) on graphene.^[^ ^92^ ^]^ Upon graphene adsorption, HEWL experienced negligible structural changes, indicating graphene could be considered a safe material in biological environments.

Studying enzyme immobilization on 2D GNMS advances their use as enzyme nanocarriers in various biocatalytic, biosensing, and biotechnological applications.^[^ ^360^, ^361^ ^]^ MD showed graphene to have the capacity to stabilize the structure and activity of the enzyme lactate dehydrogenase (LDH).^[^ ^362^ ^]^ In contrast, the binding affinity of LDH on carboxylated graphene was greater than on PG, resulting in structural fluctuations and modifications to the catalytic site of LDH. These findings contrast with another study,^[^ ^363^ ^]^ which reported 2D carbon sheet inhibited the activity of the enzyme acetylcholinesterase (AChE) due to the high surface hydrophobicity, while adsorption of AChE on GO led to conservation of the native enzyme conformation and activity, which demonstrated the potential of using GO for enzyme immobilization. Furthermore, the immobilization of the enzyme lipase on GO was explored using experiments and MD simulations for the design of enzyme carriers for improved lipase activity.^[^ ^106^, ^107^ ^]^ These studies showed that the adsorption and conformational change of lipase on GO surfaces improves the exposure of the enzyme's active site, suggesting there is an opportunity to tune the surface oxygen concentration of GO for improved enzyme activity. Other all‐atom MD simulation studies showed that GO possesses both thermoprotective^[^ ^103^, ^104^ ^]^ and nonprotective^[^ ^105^ ^]^ properties toward enzymes, however, this is highly dependent on the type of enzyme under investigation.

## 5 Nucleic Acid Interactions with GNMs: Cytotoxic Effects and Promising Biomedical Applications

Nucleic acids, including deoxyribonucleic acid (DNA) and ribonucleic acid (RNA), contain the genetic material found in all living cells that are responsible for storing and expressing genetic information. Theoretical modeling studies examining the interactions of nucleic acids with 2D GNMs can be useful for understanding potential cytotoxic effects of these materials as well as for the design of nucleotide/carbon‐based biomedical devices.

Due to the inherent small size of single nucleic acids, the interaction between 2D GNMs and DNA/RNA nucleobases, including guanine (G), adenine (A), thymine (T), and cytosine (C) and uracil (U) has been extensively studied using DFT calculations for the detection of DNA/RNA.^[^ ^118^, ^119^, ^132^, ^140^, ^144^, ^147^, ^364^ ^]^ These studies showed the interactions between DNA/RNA nucleobases and graphene are dominated by *π*–*π* stacking interactions between the purine and pyrimidine groups nitrogenous bases and the hexagonal carbon structure of graphene. The nucleobases exhibit strong physisorption on graphene, with the binding strength typically decreasing in the order of G > A > T > C > U.^[^ ^132^, ^144^ ^]^ Moreover, the electronic fingerprints of DNA/RNA nucleobases adsorbed on graphene are also affected by the adsorbed nucleobase geometry due to the strong in‐plane dipole moments of the nucleobases, where a vertical alignment of nucleobases bound to graphene has been suggested to enhance the DNA/RNA sensing capacity for graphene.^[^ ^118^ ^]^

**Figure 11:**
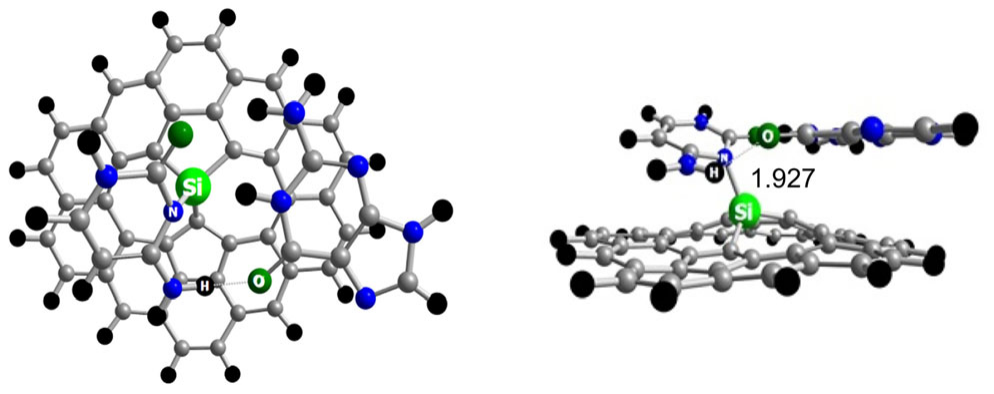
Representative DFT optimized structure of adenine–uracil nucleobase pair on silicon‐doped stone‐Wales defective graphene sheets showing the Si…N interaction. Adapted with permission.^[^ ^366^ ^]^ Copyright 2020, Elsevier.

Computational research efforts have focused on tuning the surface design of PG to enhance its binding of nucleobases to optimize its biomedical performance. For instance, DFT calculations showed GO exhibits stronger binding energy for nucleobases than PG.^[^ ^140^ ^]^ More recently, chemically doped graphene, such as titanium (Ti)‐doped graphene, was found to enhance the electrical conductivity of the material during the adsorption of thymine, suggesting greater sensing performance of doped graphene compared to pure graphene.^[^ ^119^ ^]^ Using electronic structure calculations, Mudedla et al.^[^ ^365^ ^]^ showed that the stability of modified nucleobases was highest on Si‐doped graphene compared to graphene, boron (B)‐doped graphene, and nitrogen N‐doped graphene, due to the electrostatic and covalent Si…O(N) interaction, in addition to the *π*–*π* stacking interactions. Saravanan et al.^[^ ^366^ ^]^ used QM calculations to investigate the binding strength of DNA/RNA base pair on defective and defective‐dopant graphene nanosheets. Si‐doped defective graphene significantly increased the base pair adsorption strength compared to nondoped defective and B‐doped defective graphene nanosheets. Specifically, the adsorption strength of GC base pairs on Si‐doped defective graphene was the highest, followed by AT, and finally AU base pairs. The strong base pair binding to Si‐doped defective graphene was found to be due to the partially electrostatic and covalent Si…N interaction as well as *π*–*π* stacking interactions (**Figure** **11**),^[^ ^366^ ^]^ in agreement with previous findings on pristine Si‐doped graphene.^[^ ^365^ ^]^

Other DFT studies investigated the interactions between doped graphene and modified nucleobases.^[^ ^364^, ^367^ ^]^ In one study,^[^ ^364^ ^]^ the effects of Si‐doped graphene and carboxyl‐functionalized graphene on the adsorption energy of normal (GC and AT) and caffeine‐modified DNA base pairs were explored. The study revealed DNA base pair adsorption was most favorable on carboxyl‐functionalized graphene, followed by Si‐doped graphene, and lastly, pure graphene. In another study,^[^ ^367^ ^]^ porous graphene, N‐doped, and B‐doped porous graphene were examined as sensors for epigenetically modified DNA nucleobases (5hmc, 5caC, and 5fc). The adsorption energy results identified all modified nucleobases were most strongly adsorbed on B‐doped porous graphene compared to the other particles due to the interaction affinity between the empty boron *p*‐orbitals of B‐doped graphene and the partially filled *p*‐orbital of nitrogen and oxygen atoms in the nucleobases. These findings indicated the potential biosensing application of B‐doped porous graphene. As shown in the studies above, chemical modifications of PG via atom doping provide promising benefits for the design of new graphene‐based nucleic acid sensing devices with greater detecting capacity.

MD simulations of single‐ and double‐stranded DNA (ssDNA and dsDNA) interacting with graphene and GO showed that the adsorption process is dominated by vdW and *π*–*π* stacking interactions, as well as hydrogen bonding and electrostatic interactions on GO.^[^ ^121^, ^122^, ^123^, ^141^ ^]^ Preferential binding of ssDNA to the oxidized regions of GO was observed, followed by the formation of nucleobase–GO *π*–*π* stacking interactions.^[^ ^123^ ^]^ ssDNA was found to assume a flat orientation on both GO and graphene,^[^ ^121^ ^]^ while dsDNA has been found to be oriented in both a flat and upright position on the graphene‐based surfaces.^[^ ^121^, ^141^ ^]^ The upright position of DNA on GO was driven by strong electrostatic repulsion between the negatively charged phosphate groups on DNA and oxygen atoms on GO, with the repulsion increasing in longer DNA chains.^[^ ^122^ ^]^ Moreover, all‐atom MD simulations showed that RNA hairpins interact with graphene in both unfolded and folded conformations, where the interactions were driven by hydrophobic forces between the RNA bases and the graphene surface.^[^ ^368^ ^]^

All‐atom MD simulations also showed that graphene is capable of disrupting the structures of nucleic acids due to the formation of strong *π*–*π* stacking interactions.^[^ ^124^ ^]^ The influence of graphene defects and wrinkles on adsorbed DNAs was explored using all‐atom MD simulations and revealed that graphene defects and wrinkles induce greater structural disruption and unwinding of dsDNA than on pure graphene, due to stronger base adsorption at the defect edges and wrinkled area.^[^ ^127^, ^128^ ^]^ Furthermore, wrinkled graphene has been shown to enhance the DNA sensing capacity of the graphene‐based biosensor device.^[^ ^369^ ^]^

Tuning the intrinsic/inherent factors such as the DNA/RNA sequence, as well as external factors, such as pH, temperature, and solvent characteristics, aids the design of DNA/RNA−graphene biomedical devices with specific functionalities. For example, poly‐cytosine (poly‐C) DNA has been found to attain high binding affinity to GO than other DNA homopolymers.^[^ ^370^ ^]^ Using a combination of experiments and all‐atom MD simulations, Lopez et al.^[^ ^149^ ^]^ examined the interactions between DNA homo‐oligomers and GO at varying pH, aiming to identify the reason behind the different binding affinity of DNA sequences to GO. The experiments and MD simulations showed poly‐C DNA adsorption on GO was most favorable compared to poly‐A and poly‐T DNA adsorption at neutral pH. The MD simulations helped explain the different adsorption affinities by showing that poly‐C DNA promoted greater hydrogen bond formation between the phosphate backbone of poly‐C DNA and GO compared to the other DNA homo‐oligomers, resulting in a more flexible and extended poly‐C DNA adsorbed conformation.

The delivery of short‐interfering RNA (siRNA) duplex carried by 2D platforms to an infected human cell for gene silencing has promising therapeutic potential.^[^ ^371^, ^372^ ^]^ Unzipping of the nucleic acid substrate on the nanocarrier is an important mechanism that allows for the therapeutic action of siRNA. Using DFT and all‐atom MD simulation, Mogurampelly et al.^[^ ^373^ ^]^ showed that double‐stranded siRNA spontaneously unzips and binds to graphene, leading to the formation of a siRNA–graphene complex with promising applications for siRNA delivery through lipid bilayer penetration and the disassociation of single RNA chains for gene silencing. Another observation was the reduced unzipping capacity of dsDNA on graphene compared to siRNA, where the strong interaction between uracil and graphene contributed to the siRNA unzipping. However, a limitation of chemically unmodified nucleic acids such as siRNA is their instability in human serum due to enzyme degradation.^[^ ^374^ ^]^ Thus, there is an opportunity to synthesize non‐natural nucleic acid analogs with improved stability and function in biological milieu for therapeutic applications. For example, xylonucleic acid (XNA), an RNA duplex analog containing a potentially prebiotic xylose sugar in its backbone,^[^ ^375^ ^]^ is a promising candidate for gene detection and therapeutics due to its considerable stability in human serum. Using atomistic MD simulations, Ghosh and Chakrabarti^[^ ^129^ ^]^ observed graphene unzipped XNA quicker than native RNA, due to the weakening of the Watson−Crick (WC) base pairing in XNA compared to RNA, suggesting the XNA−graphene delivery system could be a suitable candidate for inhibiting gene expressions. Recently, the influence of temperature on the flexibility of graphene and the unzipping of RNA duplex adsorbed on graphene was studied using atomistic MD simulations.^[^ ^376^ ^]^ The unzipping of RNA duplex during graphene adsorption was more pronounced on unrestrained (flexible) graphene compared to fixed (frozen) graphene. These findings indicated that the flexibility of graphene can be utilized to unzip the adsorbed RNA duplex. The effects of temperature on the unzipping process of flexible graphene were also studied, elucidating that room and lower temperatures were more effective at RNA duplex unzipping on flexible graphene than at higher temperatures of 333 K.

The influence of external conditions such as pH, ionic concentration, and temperature plays an important role in the development of stable and functional biomaterials for DNA/RNA delivery. The effects of different pH on DNA/RNA adsorption to GO were explored using all‐atom MD simulations.^[^ ^149^ ^]^ The results showed the adsorption energy of poly‐C DNA on GO was greater at neutral pH compared to acidic pH conditions. This was consistent with experimental observations and explained by greater DNA–GO hydrogen bonding at pH 7. In a combined experimental and atomistic MD simulation study,^[^ ^377^ ^]^ GO was observed to form a stable complex with siRNA, suggesting GO as suitable platform for the delivery of siRNA. The influence of ionic strength on the binding affinity of siRNA to GO was also investigated using MD simulations at different concentrations of NaCl (0, 150, and 200 mM). A higher concentration of NaCl promoted stronger siRNA–GO binding, likely due to the decrease in electrostatic repulsion between the negatively charged oxygen‐containing functional groups on GO and the phosphate groups in siRNA, in agreement with spectroscopy data. This suggested that the release of siRNA from the GO surface can be controlled by manipulating the specific cellular environment. More recently, using all‐atom MD simulations, Razavi et al.^[^ ^378^ ^]^ reported the effect of temperature (310, 320, and 330 K) on the adsorption of siRNA on GO, identifying 310 K promoted the most stable siRNA–GO complex due to greater hydrogen bond formation.

The large surface area of graphene suggests a possibility of its use as a scaffold to form nucleobase/DNA assemblies with biomedical potential. Using MetaD simulations, Spiwok et al.^[^ ^155^ ^]^ found the heterogenous nucleobase dimer assembly onto graphene was driven by graphene–nucleobase *π*–*π* stacking interactions and nucleobase–nucleobase hydrogen bonds. In another study,^[^ ^368^ ^]^ the self‐assembly of RNA molecules over graphene was examined using both experiments and all‐atom MD simulations. The simulations revealed the formation of RNA clusters on graphene, stabilized by base contacts with graphene and RNA–RNA hydrogen bonds. This finding was consistent with the RNA clusters on graphene observed in AFM experiments in a dilute RNA solution. Other MD simulation studies demonstrated that the concentration of nucleobases^[^ ^152^, ^153^, ^154^ ^]^ and the presence/absence of water^[^ ^152^, ^153^ ^]^ both influence the assembled aggregate pattern and morphology over graphene, demonstrating there is opportunity to tune the nucleic acid assembly process by controlling the external conditions.

**Figure 12:**
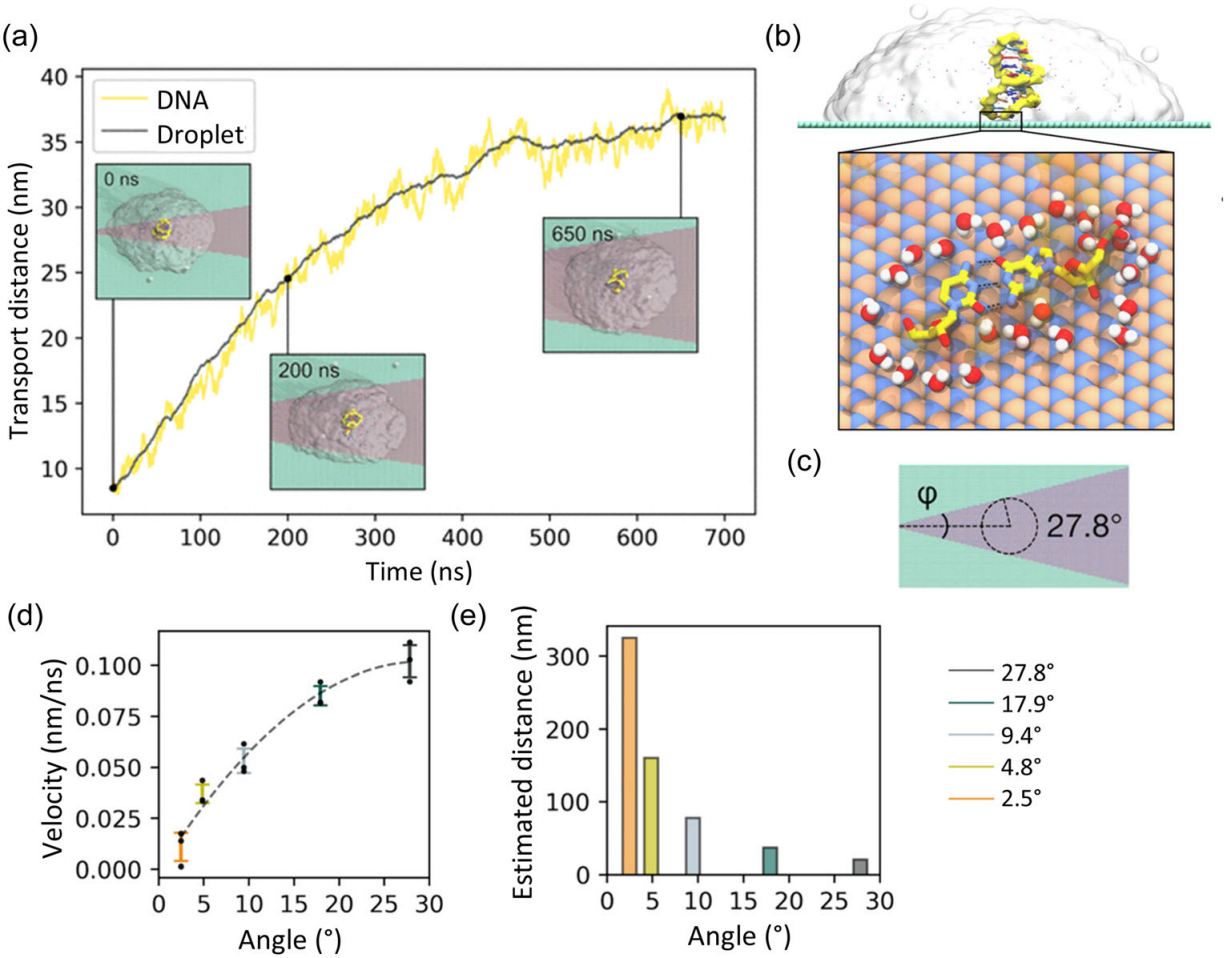
Spontaneous transport of DNA through a water droplet over a patterned 2D graphene–hBN heterostructure. a) Center of mass (COM) distance of the droplet (black line) and DNA (yellow) with respect to time. b) Adsorbed conformation of double‐stranded DNA (dsDNA) in the droplet over the graphene–hBN surface, highlighting the interaction between the two DNA bases and hBN. c) Graphene–hBN heterostructure schematic illustrating the definition of the apex angle of the hBN isosceles triangle. Various heterostructures were created with varied apex angle (27.8, 17.9, 9.4, 4.8, and 2.5°) to investigate the impact of pattern geometry on nanodroplet transport. d) Average velocity of the droplets on the graphene–hBN surfaces varying in apex angle. e) Maximum travel distance of the droplets on the graphene–hBN surfaces varying in apex angle. Adapted with permission.^[^ ^380^ ^]^ Copyright 2024, American Chemical Society.

In addition to DNA/RNA self‐assembly, the large surface of 2D GNMs can also be exploited as surfaces for the transport of biomolecules in nanodroplets. The directional transport of biomolecules is useful in the design of biomedical devices which rely on microfluidic/nanofluidic systems.^[^ ^379^ ^]^ A promising approach of biomolecular transport involves the self‐propelled directional movement of a water droplet containing biomolecules on a patterned surface. This concept was explored in a recent all‐atom MD simulation study by Xie et al.^[^ ^380^ ^]^ who proposed a patterned 2D graphene–hBN heterostructure for the spontaneous movement of a nanodroplet carrying DNA, RNA and peptides (**Figure** **12**). The simulations showed the spontaneous transport of nanodroplets containing the nucleic acid, with the self‐propelled motion driven by the differential surface chemical potential of hBN and graphene (Figure 12a). The authors ran additional simulations with different sizes of hBN by adjusting the apex angle of the isosceles hBN triangle, resulting in understanding into tuning the transport speed and distance by the sheet design (Figure 12c–e). Specifically, reducing the apex angle resulted in longer‐distance transport due to reduced transport speed (Figure 12d,e). The simulations showed that the self‐propelled transport of DNA and RNA through a water droplet over the 2D graphene–hBN heterostructure did not affect secondary structure of the biomolecules. Further, hydrophobic interactions between the bases were driving the adsorption due to the formation of surface–base *π*–*π* stacking interactions (Figure 12b). These molecular insights support the promising applications of 2D graphene–hBN heterostructures to enable a spontaneous and directional transport of DNA/RNA in various biomedical applications.

**Figure 13:**
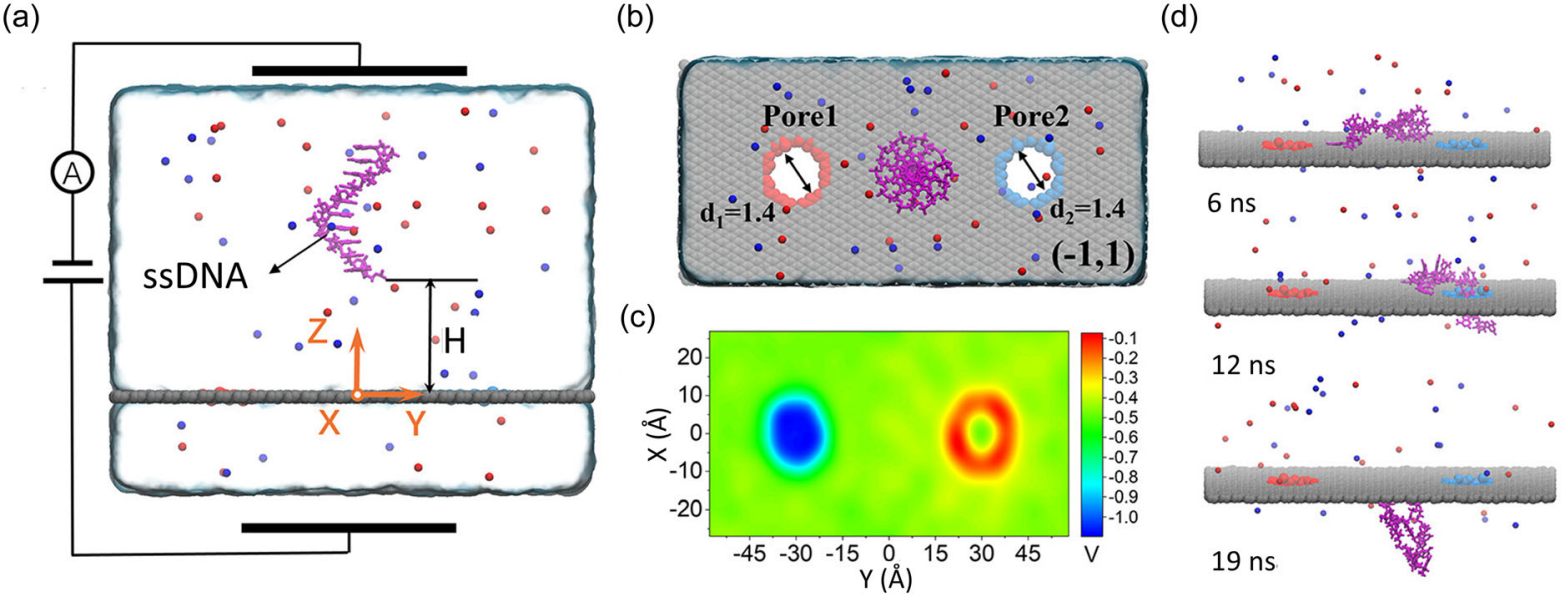
DNA transport through a charged double graphene nanopore system. a) Side view schematic of the system containing ssDNA (purple), a graphene nanopore (gray), potassium ions (blue), chloride ions (red), and the ionic solution (blue). b) Top view schematic of the system illustrating details of the graphene nanopore, including two pores of the same diameter and different charge. The ssDNA molecules are placed between the two nanopores. c) 2D potential distribution of nanopore on the X–Y plane under an electric field. d) Simulation snapshots showing the time evolution of ssDNA through the nanopore. Adapted with permission.^[^ ^384^ ^]^ Copyright 2024, American Chemical Society.

Another promising biomedical application of graphene is in its use as synthetic nanopores for the detection of genetic material.^[^ ^381^, ^382^ ^]^ These devices typically operate using an ionic current for sensing, whereby threading of DNA through a voltage‐biased nanopore induces a drop in ionic current, enabling the detection of individual bases within the DNA based on the changes in ionic current.^[^ ^383^ ^]^ Theoretical modeling has been used to investigate the capacity of graphene nanopores to detect DNA, as well as in efforts to tune the design of the nanopore for optimal sensing (see ref. [64] and references therein). Recently, explicitly solvated MD simulations were used to investigate the control of DNA transport across graphene nanopores by tuning the surface charge density and size of the nanopore (**Figure** **13**).^[^ ^384^ ^]^ The neutral nanopore promoted random ssDNA nanopore translocation, whereby ssDNA could enter either pore. However, when one pore was negatively charged and the other positively charged, this combination promoted higher and more accurate ssDNA translocation through the positively charged nanopore (Figure 13d). This was explained by the complementary attractive forces between the negatively charged ssDNA and the positively charged pore which was able to draw in the ssDNA. The negatively charged pore induced electrostatic repulsion which enhanced the selectivity of the positively charged pore. This combination of charged systems, albeit with different‐sized nanopores, was also capable of capturing and translocating ssDNA through the graphene membrane.

### 5.1 Modeling Considerations and Challenges

Accurately modeling the interactions between 2D GNMs and nucleic acids requires the development of FFs that reproduce experimental binding data. Ranganathan et al.^[^ ^143^ ^]^ employed a combination of experimental binding data and DFT calculations of nucleosides and oligonucleosides binding to GO/graphene to develop a MM FF that accurately captures the interactions between ssDNA and graphene surfaces in water. Another important consideration in studying nucleobase–graphene interactions using empirical FFs is the inclusion of polarizability, as highlighted by a recent all‐atom MD simulation study.^[^ ^150^ ^]^ The inclusion of polarizability in the FF led to significant differences in the interaction pattern of nucleobases with graphene and results closely resembling that of experiments compared to the results from nonpolarizable FF simulations. Specifically, the inclusion of polarization was necessary to capture the formation of nucleobase–graphene *π*–*π* stacking interactions and hydrogen‐bonded network leading to the formation of higher‐order structures. In a subsequent study,^[^ ^154^ ^]^ the polarizable FF was then used to study the self‐assembly of cytosine nucleobases on graphene at different cytosine concentrations. The accurate description of nucleobase–graphene interactions (*π*–*π* stacking and hydrogen bonds) by the polarizable FF was important in exploring the concentration‐dependent spontaneous assembly of cytosine nucleobases on graphene, providing new molecular insights into nucleobase self‐assembly that were not captured by nonpolarizable FF simulations.

## 6 Membrane Interactions with GNMs: Cytotoxic Effects and Promising Biomedical Applications

Classical MD simulations, including both all‐atom and CG representations, have been applied to study the interactions between 2D GNMs and biological membranes, providing insights into the potential toxicity of these materials in the cellular environment. Planar lipid bilayers are typically used as a simplified model of the cell membrane, comprised of homogenous or heterogenous lipid molecule types with hydrophilic lipid heads and hydrophobic lipid tails.

**Figure 14:**
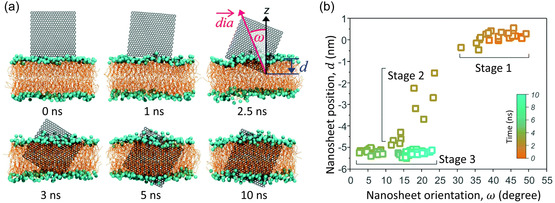
Graphene penetrating through a phospholipid bilayer. a) Simulation snapshots showing the membrane penetration pathway of graphene beginning at graphene's edges. b) Relationship between nanosheet orientation and position inside the membrane, shown in (a), demonstrating the three stages of the membrane insertion process. Adapted with permission.^[^ ^179^ ^]^ Copyright 2022, The Royal Society of Chemistry.

One of the cytotoxic mechanisms of 2D GNMs is their destruction of biological membranes through membrane insertion and lipid extraction, as evidenced in many molecular simulation^[^ ^168^, ^172^, ^173^, ^174^, ^176^, ^177^, ^178^, ^319^ ^]^ and experimental studies.^[^ ^169^, ^385^ ^]^ MD simulations also showed that the entry of graphene and GO into lipid bilayers is initiated by the edges and corners of the nanosheet (**Figure** **14**).^[^ ^169^, ^176^, ^178^, ^179^, ^386^ ^]^ Studies revealed the effects of 2D GNMs on cell membranes can be controlled by the surface design features of the graphene‐based material. For instance, in vacuo CG MD simulations showed larger graphene nanosheets (30 × 30 nm^2^) disassembled and extracted lipids from a membrane model bilayer more severely than smaller‐sized nanosheets (10 × 10 nm^2^).^[^ ^167^ ^]^ All‐atom MD simulations examining the interactions between graphene/GO and 1‐palmitoyl‐2‐oleoyl‐*sn*‐glycero‐3‐phosphocholine (POPC) lipid bilayers showed that graphene was able to penetrate into the internal apolar bilayer region, while lipid–GO hydrogen bonding at the top leaflet prevented the penetration of GO into the inner membrane.^[^ ^387^ ^]^ Similar findings were observed in an earlier all‐atom MD simulation study considering 1,2‐dipalmitoyl‐*sn*‐glycero‐3‐phosphorylcholine (DPPC) bilayers, where spontaneous insertion of GO into the bilayer did not occur within the simulation timeframe, while for graphene, the insertion into membrane was immediate.^[^ ^174^ ^]^ In contrast, using CG MD simulations, a separate study showed that larger and more oxidized graphene NFs were able to penetrate membranes slower than PG and smaller NFs.^[^ ^176^ ^]^ Specifically, PG spontaneously penetrated the membrane within a fraction of a microsecond, while GO required more than a microsecond for membrane penetration. This suggests that longer simulation timescales are necessary to observe GO insertion, and these timescales were not reached in the all‐atom MD simulation studies that reported contradictory results of GO insertion.^[^ ^174^, ^387^ ^]^

Surface coatings composed of ssDNA,^[^ ^388^ ^]^ BSA,^[^ ^69^ ^]^ and lipids^[^ ^178^ ^]^ were used to reduce the cytotoxic effects of graphene/GO. MD simulations suggested that such coating could be able to reduce or prevent the membrane destructive capacity of the graphene nanoparticles by weakening their interactions with the lipid molecules. For example, the ssDNA‐coated graphene was theoretically found to reduce the penetration depth of graphene through the phospholipid bilayer by disrupting the hydrophobic graphene–membrane interactions responsible for membrane penetration.^[^ ^388^ ^]^ Interestingly, while MD simulations showed surface passivation of GO with PEG chains resulted in incomplete insertion of the nanosheet into the membrane, experiments demonstrated that this adsorption mode may still amplify the interactions between GO and stimulatory surface receptors, indicating that immunological responses may not be avoidable by only using surface passivation.^[^ ^181^ ^]^

In light of the experimental evidence showing toxicity of GNMs through inhalation,^[^ ^389^ ^]^ Luo et al.^[^ ^390^ ^]^ applied CG MD simulations to understand the interactions between 2D GNMs, including graphene, GO, and curved graphene nanosheets, and a pulmonary surfactant (PS) layer, which is the first barrier between NPs and the deep lung after particle inhalation. The PS model was comprised of water slab with two symmetric PS layers containing DPPC, 1‐palmitoyl‐2‐oleoyl‐*sn*‐glycero‐3‐[phospho‐*rac*‐(1‐glycerol)] POPG, cholesterol molecules, and hydrophobic PS‐associated proteins, at the water–air interfaces. The graphene nanosheets were found to extract PS molecules from the layer, resulting in the formation of inverse micelles. The extraction process and micelle assembly pattern were influenced by the nanosheet size, oxidation degree, and curvature. While the formed PS corona over the nanosheets can be expected to interact with other biomolecules within the lungs, the MD simulations suggested the toxicity of GNMs through inhalation be associated with the PS lipid extraction and the resultant depletion of the PS barrier.

In contrast, the ability of 2D GNMs to disrupt the structural integrity of cell membranes, as highlighted above, can be exploited as an advantage for the design of graphene‐based biomedical devices able to destroy pathogenic microorganisms. There is substantial evidence to suggest that graphene exhibits promising antibacterial activity via the penetration and destruction of bacterial cell membranes.^[^ ^171^, ^184^, ^391^, ^392^, ^393^ ^]^ This mechanism was shown in an early experimental study, wherein graphene and GO nanosheets induced the degradation of cell membranes of *Escherichia coli*, and a series of all‐atom MD simulations revealed the mechanism behind the cell membrane degradation process.^[^ ^168^ ^]^ Other studies showed that the mode of interaction of 2D GNMs with bacterial cell surfaces, whether through parallel or perpendicular contacts, can be controlled by the GO's oxygen content.^[^ ^184^ ^]^ Recently, Ashari Astani et al.^[^ ^394^ ^]^ examined the interactions between graphene and different types of bacteria using all‐atom MD simulation to elucidate the molecular mechanisms behind their experimental findings. To simplify the bacterial cell wall composition, the outermost layer of the bacteria was modeled, which included the peptidoglycan network for *Staphylococcus aureus* and lipopolysaccharides for *Pseudomonas aeruginosa*. The simulations showed that graphene interacted with and entangled the peptidoglycan network, indicating potential bacterial inhibition due to the graphene interactions. However, for *Pseudomonas aeruginosa*, the lipopolysaccharides did not favorably interact with graphene, suggesting that bacterial inhibition by graphene may differ depending on the type of bacteria in consideration. Moreover, Khedri et al.^[^ ^89^ ^]^ demonstrated the potential destructive ability of GNMs toward SARS‐CoV‐2 by conducting microsecond‐long atomistic MD simulations of systems containing graphene‐based nanosheets and a DPPC bilayer, which replicated the SARS‐CoV‐2 lipid envelope that supports the virus structure.^[^ ^395^ ^]^ The simulations showed functionalized *P*‐doped graphene, followed by graphene, were best at penetrating the phospholipid membrane bilayer, indicating they can be promising therapeutic candidates capable of rupturing the viral membrane.

**Figure 15:**
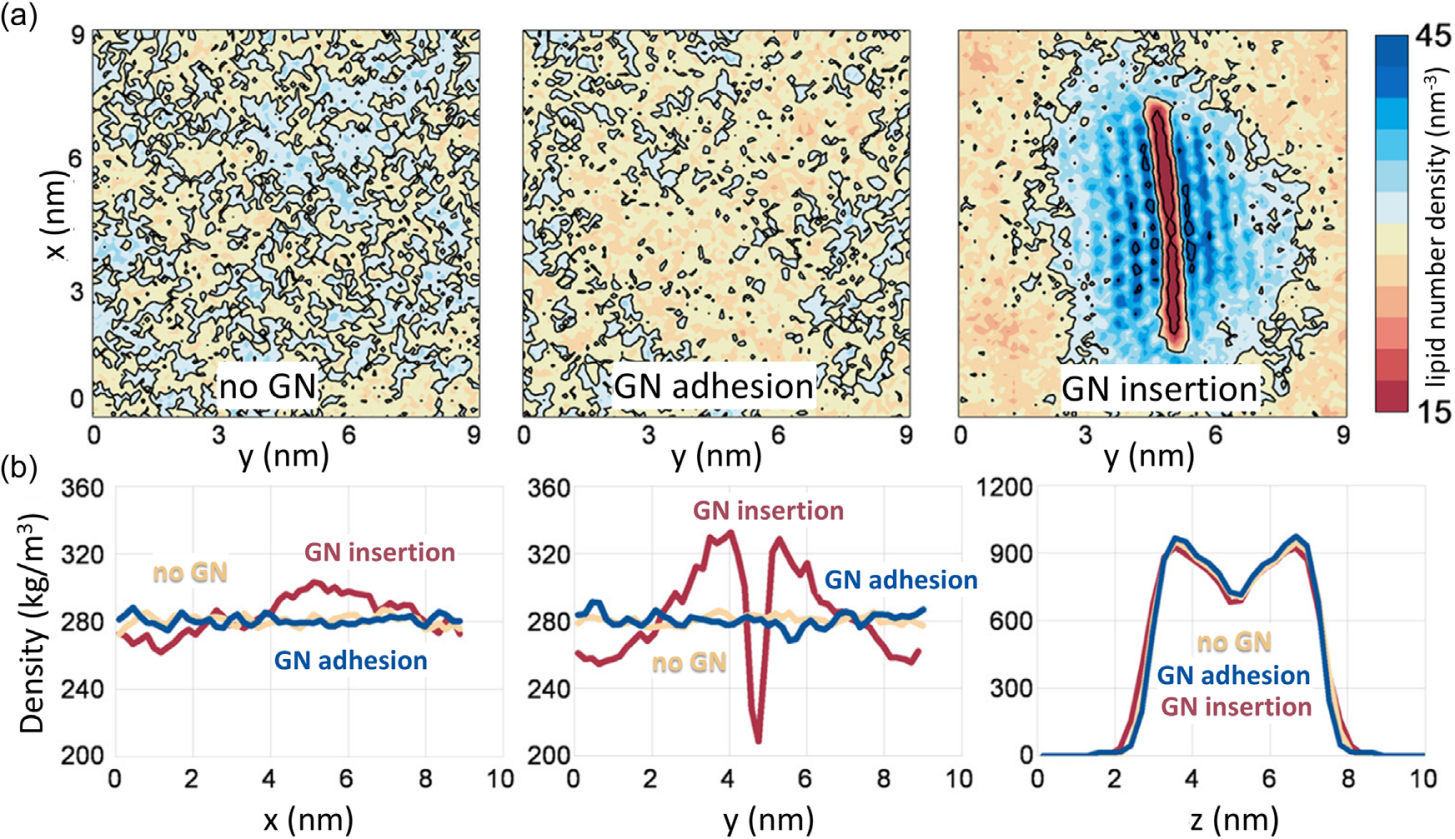
Molecular insights into the effects of graphene nanosheet (GN) insertion on the nanoscale ordering of lipids. a) 2D density maps showing the density of lipids when graphene is absent (left), adhered to the membrane (middle), or inserted into the membrane (right). b) Mass density profiles of lipids along the *x*‐, *y*‐ and *z*‐axis. Reproduced with permission.^[^ ^396^ ^]^ Copyright 2020, American Chemical Society.

Another promising biomedical application of 2D GNMs includes their potential to control or repair lipid partitioning, thereby preserving the asymmetrical composition of membranes important for cellular function and disease prevention. Atomistic MD simulations showed that graphene inserted or adhered to a POPC bilayer can reduce the rate of lipid flip‐flop transitions.^[^ ^396^ ^]^ For inserted graphene, this occurs due to formation of a nanoscale lipid‐ordered domain ≈4 nm in size, involving closely packed hydrocarbon chains, as shown by the dotted patterns in the 2D density maps (**Figure** **15**a) and a spike in the density of POPC lipids along the *x*‐ and *y*‐axis (Figure 15b). The lipid‐ordered domain was found to increase the energy barrier for lipid flip‐flop, as shown by the PMF obtained from the US simulations. Recently, Zhu et al.^[^ ^397^ ^]^ applied all‐atom MD and US simulations to examine the differences in graphene and GO in mediating lipid flip‐flop transitions. Consistent with the previous study,^[^ ^396^ ^]^ graphene was found to decrease lipid flip‐flop rate due the formation of an ordered nanodomain hindering the migration of nearby lipids across the membrane. In contrast, GO was observed to increase the rate of lipid transport by inducing the formation of water pores which increased lipid scrambling. Thus, while graphene may act as an inhibitor of lipid translocation, GO could be a promising candidate to regulate lipid partitioning in cellular membranes.

**Figure 16:**
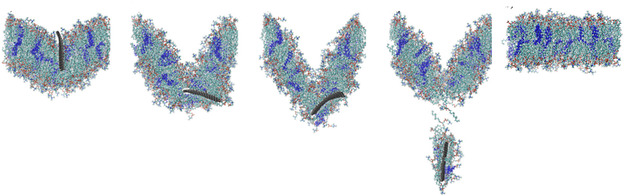
SMD simulation snapshots at different time intervals showing the membrane indentation, withdrawal, and self‐healing process during interaction with graphene. Reproduced under terms of the CC‐BY 4.0 license.^[^ ^398^ ^]^ Copyright 2020, The Authors. Published by American Chemical Society.

Molecular simulation insights into the mobility/movement of graphene through lipid bilayers can provide understanding of the performance of 2D GNMs for drug delivery applications. Recently, SMD simulations were used to study the insertion and removal of graphene nanosheets of different sizes into a bilayer, aiming to understand the potential use of 2D GNMs as phospholipid bilayer nanoindenters for targeted drug delivery (**Figure** **16**).^[^ ^398^ ^]^ Simulated indentation and withdrawal of graphene from the bilayer were achieved by adding virtual springs to the atoms located on the sides of the graphene nanosheet models and adding a force to move the nanosheet through the bilayer at a rate of 1 and 2 m s^−1^. Through this simulated process the membrane experienced structural changes and lipid extraction which was most extreme for the systems containing larger nanosheets. However, following initial membrane destruction, structural regeneration (membrane recovery) was also observed through the removal of water molecules introduced by the indentation process. These simulation findings suggested that graphene could be considered as a plausible candidate for nanoindentation in drug delivery applications due to its ability to effectively move through the nanosheet and prevent permanent membrane damage. In another study,^[^ ^378^ ^]^ atomistic MD simulations were used to investigate the spontaneous diffusion of GO complexed with siRNA into a POPC–cholesterol lipid bilayer for the design of a GO‐based siRNA delivery system. The ability of the siRNA–GO complex to cross the cell membrane suggested a possibility of siRNA delivery using the GO complex‐based device.

Understanding the molecular organization and stability of lipid membranes on graphene‐based surfaces can facilitate the development of biosensing and drug delivery devices based on supported phospholipid membranes.^[^ ^399^, ^400^ ^]^ CG MD simulations were used to study the organization of lipid bilayers on graphene and GO, revealing differing lipid topologies on each surface, including the disassembly of an inverted bilayer on graphene and, a bicelle‐like configuration on GO stabilized by interactions between lipid heads with the surface and solvent.^[^ ^167^ ^]^ To gain insights into the structure and dynamics of lipids adsorbed onto graphene, Rivel et al.^[^ ^401^ ^]^ studied mono‐, bi‐, and trilayers of DOPC lipids deposited on graphene using all‐atom MD simulation in the presence and absence of water. The lipid molecules were observed to organize into various structures, including homogenous lipid layers, networks of inverted micelles, and cylindrical monolayer‐bound micelles. The lipid organization process was driven by the matching of hydrophobic and hydrophilic interfaces between graphene, the lipid layers, and water. More recently, the effect of surface hydrophilicity on supported lipid bilayers was investigated using all‐atom MD simulations of dioleoylphosphatidylcholine (DOPC) bilayers placed on graphene and GO surfaces.^[^ ^402^ ^]^ Lipid‐graphene systems varying in GO oxidation levels (0, 5, and 17%), ratio of epoxy‐to‐hydroxyl groups (0:0, 1:1, and 2:1), lipid model type (infinite bilayer and a bicelle) and amount of water between the lipid‐surface interface (dry or wet), were modeled using a series of MD simulations. The hydrophilicity of the GO surface was observed to influence the bilayer thickness, lipid orientation, and the extent of surface hydration. The inhomogeneous GO surface resulted in partitioning of lipid tails on the hydrophobic pure carbon regions, while hydrophilic lipid heads favored contacts with the hydrophilic oxygen‐rich regions. Using all‐atom and CG–MD simulations, the curvature of graphene was also shown to influence the organization and morphology of DOPC lipids.^[^ ^403^ ^]^ The MD simulations revealed sinusoidally crumpled graphene impacted the adhesion strength of the lipids, leading to different structural and dynamic properties in the supported lipid monolayer. Specifically, concavely curved graphene promoted stronger adhesion of lipid tails compared to convexly curved graphene, due to the more ordered lipid tails in the concave region. These findings correlate with an earlier all‐atom MD simulation study which found concave graphene promoted stronger extraction of lipids than flat or convex surfaces.^[^ ^170^ ^]^

### 6.1 Modeling Considerations and Challenges

An important consideration when using theoretical modeling techniques to predict the behavior of NMs in biological environments is the accuracy of the cell membrane model. This was addressed by Puigpelat et al.^[^ ^176^ ^]^ who included cholesterols and differently ordered lipid phases to their CG membrane models, providing a more accurate representation of graphene interactions and insertion mechanisms into biological membranes. The more complex membrane model was found to influence the orientation and mobility of inserted graphene, resulting in more tilted graphene orientations and reduced mobility of the inserted graphene. Furthermore, the favorable interactions between graphene and the heterogeneous lipid phases were impacted. Specifically, inserted graphene avoided interactions with cholesterol molecules, leading to graphene migration to the disordered phases of the membrane with reduced cholesterol content. In another CG MD simulation study, Santiago and Reigada^[^ ^386^ ^]^ observed graphene to favor a perpendicular or tilted orientation in a liposome‐cholesterol bilayer to maximize contacts with phospholipid tails and avoid contacts with cholesterol molecules. By using liposomes as the membrane model, the effects of membrane curvature on the orientation of inserted graphene were identified. Specifically, membrane curvature was found to prevent the complete coverage of graphene within the two leaflets, as opposed to the complete coverage of graphene observed in planar membrane models.

The bilayer lipid composition can also influence the interaction and insertion of graphene in membrane models. This was demonstrated in a recent study using all‐atom MD simulations investigating the penetration of graphene into bilayers varying in phospholipid headgroups.^[^ ^179^ ^]^ Out of the different headgroups studied (PA, PE, PG, PI, or PS), graphene insertion into PI and PS bilayers was most difficult due to strong interactions between adjacent lipid headgroups that prevent the nanosheet edge from piercing through the membrane.

The presence of cholesterol is known to affect the structural lability of biological membranes. The effects of cholesterol concentration (0–40%) in POPC bilayers on the interactions with graphene and GO were explored using all‐atom MD simulations.^[^ ^387^ ^]^ Increasing the concentration of cholesterol in the lipid bilayer resulted in faster membrane insertion of graphene compared to systems with pure POPC. In the simulations with GO, the inclusion of cholesterol in the bilayer led to lipid disruption only at the POPC–GO binding interface. Thus, the inclusion of cholesterol in the lipid bilayer model affected the membrane‐destructive effects of the GNMs. More recently, all‐atom MD simulations were used to examine the influence of cholesterol on the interactions between graphene and DPPC bilayers.^[^ ^404^ ^]^ In this study, it was theoretically shown that in the absence of cholesterol, graphene penetrated the bilayer and remained vertically inserted, while in the membrane with 20% cholesterol, graphene remained inside the bilayer in a parallel orientation. The cholesterols were observed to preferentially adsorb on graphene which led to a disturbance in the local order of the DPPC membrane. These findings indicate that the potential toxic effects of graphene are likely to depend on the membrane's cholesterol concentration.

## 7 Recent Progress in Understanding the Toxicity of 2D GNMs

The toxicity and safety of NMs for humans and the environment are critical issues concerning their proliferation in biomedical applications. While the previous sections demonstrated a great potential for 2D GNMs in medicine, including diagnostics, therapeutics, and drug delivery due to the unique combination of physical and chemical properties, understanding their biosafety is essential for safe and effective implementation in health‐related applications.

Experimental studies have advanced our understanding of the GNM toxicity through in vitro and in vivo experiments across various bacterial, fungal, and mammalian cells, as well as in the animal models. These techniques provided real‐time information on the effects of GNMs on different organ systems, including insights into short‐ and long‐term toxicity, accumulation, biodegradation, and excretion of the NMs following exposure. Experimental studies investigating the impact of 2D GNMs on health, including effects on the skin, immune, pulmonary, cardiovascular, nervous, reproductive, and developmental systems, as well as on the gastrointestinal tract, liver, spleen, and kidneys, were comprehensively reviewed in numerous publications over the last decade (see reviews^[^ ^23^, ^24^, ^25^, ^26^, ^27^, ^389^, ^405^ ^]^ and references therein). Although this review primarily focuses on the theoretical modeling, we provide a summary of key experimental findings on toxicity, given the crucial role of experiments in enhancing our understanding of GNM toxicity and interpretation/validation of simulation‐acquired data.

Previous studies have demonstrated that toxicity of 2D GNMs is linked to their ability to enter cells by penetrating lipid membranes,^[^ ^169^, ^385^ ^]^ aligning with the modeling findings presented in Section 5 and 6. This capability can, however, be advantageous for the destruction of harmful cells, including cancerous, bacterial, and fungal cells.^[^ ^392^, ^406^, ^407^, ^408^ ^]^ Substantial evidence indicates that the toxicity of 2D GNMs depends on the dosage as well as the exposure time, as seen by cell‐ and animal‐based experiments.^[^ ^385^, ^409^, ^410^, ^411^ ^]^ Furthermore, experiments demonstrated that since the physicochemical properties of 2D GNMs impact their toxicity, their biocompatibility and biosafety can be improved^[^ ^412^, ^413^, ^414^ ^]^ or worsened^[^ ^415^, ^416^ ^]^ through surface functionalization. In addition to surface functionalization, the size of 2D GNMs is another factor influencing their toxicity. An experimental study found that larger GO sheets were more destructive to bacterial cells because they provided better cell coverage, which resulted in decreased cell viability.^[^ ^417^ ^]^ Other studies observed greater cellular internalization of GO particles with smaller (circa 50–350 nm) lateral dimensions,^[^ ^418^, ^419^ ^]^ while the larger (circa 750–1300 nm) ones preferred to remain adsorbed to the plasma membrane and activate certain immune pathways.^[^ ^419^ ^]^ The size of GO nanoparticles was also found to influence their biodistribution, accumulation, biodegradation, and excretion in mice.^[^ ^420^, ^421^, ^422^, ^423^ ^]^ Another important toxicity consideration is the purity of GO, which can be compromised during synthesis and preparation processes, potentially influencing the results of toxicity assessments/outcomes. Interestingly, purification by repeatedly washing GO with warm water through centrifugation and isolating single‐layered GO from the separated product to remove slightly soluble mellitic acid resulted in purified GO that neither induced cytotoxic responses in vitro, nor caused inflammation in mice.^[^ ^424^ ^]^

The toxicity of 2D GNMs is also influenced by the translocation and fluid/organ exposure route in the body. The human entry routes of NMs include inhalation, ingestion, dermal penetration, and injection, each differing in the first point of contact with the body.^[^ ^425^ ^]^ Blood components, such as proteins, enzymes, and small organic molecules, are the first point of contact for intravenous injections, followed by their journey to other organs. A study showed the interaction between blood and GO caused thrombosis in mice by promoting platelet aggregation, highlighting the toxic effects of GO on the blood.^[^ ^426^ ^]^ Another important consideration is the alteration of GNMs by biomolecules in the blood, such as the formation of a protein corona, which can affect the biological identity of the introduced NM.^[^ ^28^, ^29^ ^]^ Notably, experiments showed that the formed protein corona on GNMs can exhibit either protective^[^ ^85^, ^427^, ^428^ ^]^ or destructive^[^ ^429^ ^]^ effects on cells. Furthermore, when the desired application of GNMs is to exploit its toxicity for anticancer or antibacterial purposes, the protein corona can negatively impact the activity of GO.^[^ ^430^ ^]^ Therefore, the exact implications of protein coronas on desired GNMs function and unintended consequences are still uncertain.

Toxicity studies focusing on the oral ingestion of 2D GNMs and their effects on the digestive tract highlight their safety for applications in food packaging and pharmaceuticals. For example, the oral administration of PEGylated GO derivatives showed no tissue uptake, limited intestinal adsorption, and rapid excretion in mice.^[^ ^431^ ^]^ However, some studies suggest that GNMs do not undergo detectable structural degradation following digestion,^[^ ^432^ ^]^ while others showed that the digestive process may cause significant morphological changes, such as GO nanosheet folding.^[^ ^433^ ^]^ However, in both cases, the digested GNMs did not damage the intestinal cells between one and nine days post exposure.^[^ ^432^, ^433^ ^]^ Conversely, while no immediate cytotoxic effects of GO and graphene nanoplatelets on human intestinal barrier cells were observed in an in vitro model, these GNMs were found to induce DNA damage, raising concerns about their genotoxicity.^[^ ^434^ ^]^ Further, recent studies involving the oral administration of GO in mice found that the material can affect the ultrastructure of the intestine, alter the structure of the gut microbiota, and influence the abundance and type of antibiotic‐resistance genes.^[^ ^435^, ^436^ ^]^

Assessing skin exposure to 2D GNMs is crucial, as it is considered one of the most significant exposure routes in industrial and technological applications and informs their therapeutic use in topical creams. Importantly, the topical application of graphene and GO particles was shown to not induce toxic effects and skin sensitization,^[^ ^437^, ^438^, ^439^, ^440^ ^]^ and can even possess wound‐healing properties.^[^ ^439^ ^]^

Other studies examined the ocular toxicity of 2D GNMs and found that GO and hydroxylated GO did not cause severely harmful effects on eye health.^[^ ^441^, ^442^ ^]^ However, these materials may cause damage to eye epithelial cells if administered in large amounts for long periods of time.^[^ ^442^, ^443^ ^]^ These findings suggest that repeated GO contact with surface‐level organs like the eyes in occupational and environmental settings necessitates the use of suitable eye protection. In contrast, another study found that GO caused ocular toxicity in mice, while rGO showed no adverse ocular effects.^[^ ^444^ ^]^

Many studies examined the toxicity of 2D GNMs on the cells and tissues of the liver, spleen, lungs, and brain. For example, multiple studies showed that GO‐induced liver damage in rodents.^[^ ^445^, ^446^ ^]^ Others showed that GO triggered lung inflammation and injury in mice,^[^ ^447^, ^448^ ^]^ and damage to the liver, spleen, kidney, and testicles following respiratory exposure.^[^ ^449^ ^]^ Conversely, a recent study emulated human‐like lung exposure to GO by developing a lung organoid exposure model and found GO was not toxic.^[^ ^450^ ^]^ Regarding neural system health, an in vivo study found that oral administration of rGO in mice caused no long‐term effects on learning and memory behaviors.^[^ ^451^ ^]^ Additionally, a recent study showed that GO can have varying toxic impacts on different organs, exhibiting no toxic effects on neuronal cells but causing liver inflammation.^[^ ^452^ ^]^ Others investigated the ability of 2D GNMs to cross the blood–brain barrier and cause potential harm to its integrity.^[^ ^415^, ^453^, ^454^ ^]^ These studies found that GO did not adversely affect the integrity of the blood‐brain barrier (BBB),^[^ ^454^ ^]^ while PEGylated rGO was able to cross the BBB and induce toxicity.^[^ ^415^, ^453^ ^]^

There are also concerns that 2D GNMs may trigger adverse immune responses and induce allergic reactions. For example, studies showed that intravenous injection of graphene nanosheets in mice caused Th2 inflammatory responses in the lung and spleen,^[^ ^455^ ^]^ and blood exposure to GO‐induced anaphylactic reaction and death in nonhuman primates.^[^ ^456^ ^]^ Conversely, a recent study suggested that graphene particles have the potential to positively influence the immune system by modulating the behavior of macrophages.^[^ ^457^ ^]^

Understanding the accumulation, excretion, and biodegradability of 2D GNMs within the body after exposure is essential for their safe and effective use in biomedical applications. Studies reported GNMs accumulate in the spleen following injection and are excreted via the kidneys and urinary tract.^[^ ^458^, ^459^, ^460^, ^461^ ^]^ Recently, it was shown that layered graphene can accumulate in the lungs of mice for over a year following tracheal administration, and that the biopersistence of graphene is greater when administered in an acute high dose compared to repeated low‐dose exposure.^[^ ^462^ ^]^

Studies also explored how oxidizing species^[^ ^463^, ^464^ ^]^ and enzymes^[^ ^465^ ^]^ degrade 2D GNMs, providing insights into how the degradation process may introduce toxic products in the human body. In vivo studies investigated the biodegradation of carboxylated graphene and GO in mice over three to nine months and found that the degradation process occurs in the spleen.^[^ ^458^, ^461^ ^]^ However, the accumulation, excretion, and biodistribution pathway of 2D GNMs can vary depending on the size of the GNM.^[^ ^420^, ^421^, ^422^, ^423^ ^]^

Although the toxicity assessment of 2D GNMs has been thoroughly investigated using experiments, as summarized above, many discrepancies exist between the results across different studies. This is because a key challenge in understanding the toxicity of GNMs via experiments is that their toxicity levels vary depending on their preparation method, dose, lateral dimension, functionalization concentration, surface charges, layer number, purity, living organism model, exposure method, and application it is intended for. The uncertainty surrounding GNM toxicity in experiments warrants the need for fundamental understanding of the physicochemical mechanisms underlying the interactions of 2D GNMs with biological molecules in physiological environments. Consequently, theoretical modeling when used in conjunction with experimental methods should provide a more comprehensive understanding of the toxicity of 2D GNMs in living organisms. However, it is worth noting the toxicity information obtained from simulations differs from that of experiments. Molecular modeling captures bio–nano interactions at temporal and spatial resolutions (Figure 2) not achievable by experiments and thus provides complementary datasets for subsequent application of the data science algorithms via ML.

Exemplar molecular simulation studies reviewed in the previous sections determined the fundamental systematically varying properties that can affect the toxicity of graphene‐based NMs. For instance, modeling dispersibility and colloidal stability of 2D GNMs in aqueous solutions showed the ability of 2D GNMs to remain suspended in solution and avoid aggregation, a property important for their stability and low toxicity in biological fluids.^[^ ^22^ ^]^ The modeling studies examined how the properties of GNMs, such as size, functionalization, and purity level, along with external solution conditions like pH, ions, and metal cations, affect the behavior of GNMs in solution. The findings indicated GO is more stable in water than PG, owing to its increased hydrophilicity^[^ ^218^, ^219^, ^220^ ^]^ and reduced tendency to aggregate in solution.^[^ ^222^, ^223^, ^224^ ^]^ However, low pH conditions and the presence of metal cations and oxygen debris can accelerate GO aggregation.^[^ ^222^, ^227^, ^234^ ^]^

Many theoretical modeling studies found that PG induces toxic effects due to its destructive impact on the native structure and function of peptides/proteins,^[^ ^68^, ^74^, ^75^, ^76^, ^81^, ^93^, ^291^, ^292^, ^359^ ^]^ nucleic acids,^[^ ^124^, ^127^, ^128^ ^]^ and lipid membranes.^[^ ^168^, ^172^, ^173^, ^174^, ^176^, ^177^, ^178^, ^319^ ^]^ Functionalization methods, such as surface oxidation to produce GO and surface coatings using DNA, blood proteins, and lipid molecules, were identified as effective design strategies to reduce the destructive effects of 2D GNMs toward proteins,^[^ ^73^, ^294^, ^295^, ^318^ ^]^ and membranes.^[^ ^69^, ^174^, ^176^, ^178^, ^387^, ^388^ ^]^ These simulation findings align with experimental studies which showed that the biocompatibility and biosafety of GNMs is improved through surface functionalization.^[^ ^412^, ^413^, ^414^ ^]^ However, the type, amount, and patterning of oxygen‐containing functional groups can impact GO aggregation in solution^[^ ^223^, ^224^, ^225^, ^226^ ^]^ and its interactions with biomolecules,^[^ ^296^, ^317^, ^344^ ^]^ demonstrating these factors are crucial considerations for tuning GO's cytotoxicity in physiological environments. The theoretical modeling studies also explored opportunities to exploit the toxicity of 2D GNMs by disrupting the structure and function of pathogenic bacterial membranes,^[^ ^168^, ^184^, ^394^ ^]^ viral proteins,^[^ ^87^, ^352^, ^354^, ^355^, ^358^ ^]^ and mature amyloid fibrils,^[^ ^83^, ^339^, ^342^ ^]^ all of which are harmful in the body.

Moreover, studies showed that wrinkled or defective graphene induces more structural disruption to proteins and DNA,^[^ ^127^, ^128^, ^291^ ^]^ and the lateral size of 2D GNMs influences their behavior in biological environments. For example, smaller GNMs tended to aggregate more rapidly in solution compared to larger ones,^[^ ^222^ ^]^ and larger PG sheets caused more significant disruption to the native protein and membrane structures than smaller sheets.^[^ ^167^, ^317^, ^390^ ^]^

Despite advancements in understanding GNM toxicity through theoretical modeling techniques, some conflicting findings persist among the studies. The difficulty stems from the fact that simulation findings can vary based on specific biomolecules and GNM structures under investigation, as well as the simulation methods, parameters, and timescales employed. A key example of this challenge is the use of 2D GNMs for controlling amyloid fibril formation (see Section 4.2.2), as multiple modeling studies present conflicting findings on whether these materials promote or inhibit amyloidosis. Molecular simulation methods employed also potentially impact our comprehensive understanding the 2D GNM toxicity. For example, most classical MD techniques cannot capture the long‐term effects of GNMs within the large and complex organ systems of the body which encompasses biodistribution, accumulation, and excretion of the materials throughout the body, as well as the responses of GNMs to varying physiological conditions during systemic circulation.

## 8 Conclusion

We reviewed recent theoretical modeling studies employing first principles and classical MM‐based methods to provide physicochemical insights into the mechanisms by which graphene‐based NMs, namely, graphene‐based NFs and surfaces, interact with biological environments. The computational modeling helped elucidate the energetic, structural, and thermodynamic properties of 2D GNMs as they interact with water, solvated ions and small molecules, proteins, nucleic acids, and lipid membranes. The molecular level information provided by the theoretical modeling contributed to advancement of the possible applications as well as to a better understanding of potential cytotoxic effects of GNMs in biological systems. Substantial evidence suggests that the inherent hydrophobicity of pristine 2D GNMs limits their applicability in aqueous environments by promoting strong hydrophobic and *π*–*π* stacking interactions with biomolecules leading to potentially destructive effects on the native structure and function. Therefore, most current research focuses on tuning the surface features of graphene‐based NMs, including the size, shape, and functionalization, to optimize their interactions with the biological environment. GO, in particular, is proving to be a promising candidate for biomedical applications due to its surface hydrophilicity and biocompatibility. All‐atom MD simulations enabled a systematic exploration of important surface design features of GO nanoparticles including the type, concentration, and distribution of the oxygen‐containing functional groups on its surfaces and edges. Furthermore, other functionalization strategies, including the covalent and noncovalent binding of peptides, DNA, enzymes, proteins, polymers, and inorganic atoms/molecules, were also explored.

Theoretical modeling facilitated the design advancement of graphene‐based biomedical devices for the treatment and prevention of disease and infection, as biosensors and detection devices, and as nanoenzymes. Specifically, molecular simulations have been beneficial in elucidating the mechanisms imparting the (bio)functionality of 2D GNMs to 1) prevent amyloidosis by inhibiting the spontaneous unfolding and aggregation of proteins and cutting mature amyloid fibrils; 2) interfere with infection by disrupting the structure and function of virus‐related proteins and cell membranes, as well as bacterial cell membranes; 3) act as nanocarriers for delivering anticancer and antiviral drugs or other molecules with therapeutic properties; and 4) repair lipid partitioning to preserve the asymmetrical composition of membranes important for cellular functionality and disease prevention. In addition to these therapeutic uses of GNMs, theoretical modeling studies investigated the self‐assembly of biomolecules such as AAs and peptides on graphene to form nanopatterned surfaces to facilitate the development of novel biomaterials for targeted biomedical applications. Computational studies of the interactions between DNA/RNA and graphene‐based surfaces advanced the development of DNA/RNA targeting biomedical devices. Furthermore, studies examining the molecular organization and stability of lipid membranes on graphene‐based surfaces facilitated the design of devices employing supported phospholipid membranes.

While it is evident that theoretical modeling is widely accepted as a complementary approach for progressing the fundamental understanding of the biomedical potential of 2D GNMs, it is important to acknowledge the challenges these methods face for accurate and efficient property prediction for the bio–nano systems under review, and in general. First, attaining useful information from MD simulations relies on the accuracy of the structural model. As demonstrated in the reviewed studies, different structural models of 2D GNMs can be employed, including periodic (computationally restrained) or aperiodic (computationally mobile) systems. NFs are an example of aperiodic GNM models which enable insights into the effects of the surface edges, curvature, and size, on the adsorption of biomolecules, while modeling the GNM as an infinite and planar surface is deficient of such insights.

The use of more experimentally agreeable models of 2D GNMs is necessary to increase the accuracy and relevancy of the theoretical modeling simulations. For instance, the inclusion of surface defects and curvature (wrinkles) would be useful as these deformations are often created during the manufacturing and modification processes of graphene.^[^ ^235^, ^236^, ^466^ ^]^ However, the precise details on the composition and structure of GO, such as the type and distribution of oxygen‐containing functional groups, still lack the experimental certainty. While the random placement of oxygen‐containing functional groups is the most widely used approach to create structural models of GO, some models depict GO as an inhomogeneous surface with distinct oxidized and unoxidized regions.^[^ ^240^, ^241^, ^467^ ^]^ Another limitation of common theoretical models of graphene‐based NFs is the assumption of a perfect square shape, which is idealistic compared to the more irregular shape and nonuniform size observed in microscopy experiments.^[^ ^468^, ^469^ ^]^ Furthermore, accuracy of the biological system models must be also considered to achieve a realistic representation of the interfacial interactions of GNMs with biological environments.

In addition to the structural model type, accuracy of the FF parameters used to describe the bio–GNM interactions influences the reliability of the molecular simulation findings. The reviewed studies demonstrated how the interactions between 2D GNMs and biomolecules are influenced by the atomistic FF model, thus highlighting the importance of a careful FF selection and validation, as well as often needed development of new FF parameters to enable the modeling of new functionalities and improved structural predictions. It is generally accepted that the development of experimentally agreeable FFs will enhance the reliability of atomistic simulations. An important consideration in the development of realistic FFs is the inclusion of polarizability effects which play a significant role in the adsorption of water, ions, AAs, proteins/peptides, and nucleic acids onto graphene‐based surfaces. The reviewed studies showed that the adsorption capacity and structure of these molecules over GNMs vary greatly when using a polarizable FF model compared to when the polarization effects are not accounted for. However, a notable challenge lies in the development of highly transferrable polarizable FFs for different environments, which is very computationally demanding.

To access larger scale models and longer timescales, the coarse‐grain approach is commonly used. This removes the atomistic resolution, and care should be taken that the simplified models adequately reproduce the chemical and physical details important for the system's properties and performance in applications. The herein reviewed studies highlighted useful implementation of CG MD simulations to investigate large and complex molecular processes such as the aggregation of GNMs in water and the interactions of GNMs with complex biological membranes. However, it has also been demonstrated that coarse‐graining is not readily applicable to study the adsorption of proteins and nucleic acids onto 2D GNMs, due to the limitations in elucidating the secondary structure transitions caused by conformational changes that occur upon the biomolecules adsorption on surfaces.^[^ ^30^ ^]^

The reliability of molecular simulations based on classical MD is also influenced by the conformational sampling efficacy for flexible multicomponent systems such as biomolecules in solution and on surfaces/nanoparticles. While the “brute force” spontaneous MD is still widely employed to study the bio–GNM interface, the time and length scale limitations of this method reduce its ability to adequately sample complex bio–nano systems. Examples of such systems include GNM aggregation in solution, and the adsorption, self‐assembly and adsorption of AAs, peptides/proteins, nucleic acids, and lipids on 2D GNMs. This emphasizes the need for applying the enhanced sampling algorithms, such as US,^[^ ^45^ ^]^ replica exchange‐molecular dynamics (REMD,^[^ ^46^ ^]^ or REST^[^ ^47^ ^]^/REST2)^[^ ^48^ ^]^ and MetaD,^[^ ^49^, ^50^ ^]^ which are capable of overcoming the energy barriers between multiple quasi‐stable states and widen the phase space exploration for such complex systems.

Along with the physics‐based modeling methods, ML and artificial intelligence (AI) algorithms are emerging as a powerful tool for predicting the structure‐properties relations for bio–nano systems. To date, ML algorithms have been used to generate more experimentally agreeable structures of GO,^[^ ^241^ ^]^ and doped/defective graphene,^[^ ^470^ ^]^ to develop more accurate FFs to study the aqueous GO interface,^[^ ^471^ ^]^ and to predict and optimize the charge transfer properties of graphene and GO.^[^ ^472^ ^]^ AI‐based systems are also being used to predict the structure of biological molecules based on their sequence, for example, the AI‐based tools AlphaFold2^[^ ^473^ ^]^ and AlphaFold3^[^ ^474^ ^]^ developed by DeepMind for predicting the structure of proteins, nucleic acids, and small molecules. There is also promise in using AI and ML approaches to accelerate simulations and the simulation interpretation processes.^[^ ^475^ ^]^ It should be noted, however, that while AI represents a promising new tool for structure prediction, the reliability of its prediction largely depends of the size and quality of experimental and physics‐based computational data employed for its training.^[^ ^476^, ^477^ ^]^

Above all, the toxicity and safety of 2D GNMs to humans and the environment are major issues influencing their widespread use in biomedical applications. Experiments on various cells and animal models demonstrated the effects of GNMs on different organ systems, their time‐ and dosage‐dependent toxicity, and their accumulation, biodegradation, and excretion pathways. Simultaneously, molecular modeling provided atomic‐level insights into the structural and dynamic behavior of 2D GNMs in physiological environments, at an atomic level of detail current experimental methods cannot achieve. Together, these multiscale techniques are advancing our understanding of 2D GNMs toxicity. Generally, the toxicity of 2D GNMs is influenced by the dose, duration, concentration, exposure route, and physicochemical properties of the materials. Studies showed that the biocompatibility and biosafety of graphene are improved through surface functionalization, either via surface oxidation to produce GO and the use of surface coatings. However, the size of GO, as well as the type, amount, and patterning of functional groups can impact its toxicity. Thus, it is important to tailor the physicochemical properties of 2D GNMs to minimize their toxicity. Despite progress, concerns about the toxicity of 2D GNMs to the human body persist. This is because toxicity conclusions from experimental studies vary widely due to differences in preparation methods, dose, lateral dimensions, functionalization concentration, surface charges, layer number, purity, organism models, exposure methods, and intended applications. Similarly, theoretical modeling studies show variability in toxicity findings based on the structural specifications of the bio–nano system under investigation, such as the chemical composition and size of the bio–nano system, as well as the simulation methods, parameters, and timescales used. Ultimately, more rigorous toxicological screenings, using theoretical modeling and experimental methods, particularly in vivo and clinical trials, remain necessary. Other important considerations before the practical use of 2D GNMs in medical settings include understanding their environmental and ecosystem impacts upon release, as well as addressing the challenges associated with their mass production.^[^ ^478^, ^479^ ^]^

Overall, theoretical molecular modeling has proven to be a valuable complement to experiments, enhancing our understanding of potential biomedical applications and possible cytotoxic effects of 2D GNMs. Future benefits of molecular simulations will be achieved via growing complexity and realism of the structural models, enabled by the ongoing development of inter‐atomic and CG FF, enhanced sampling algorithms as well as the application of AI and ML‐based methods in conjunction with the physics‐based in silico approaches and experiments, including animal studies and clinical trials.

## Conflict of Interest

The authors declare no conflict of interest.

## Author Contributions

**Alexa Kamboukos**: conceptualization (supporting); data curation (lead); formal analysis (lead); investigation (equal); methodology (equal); visualization (lead); writing—original draft (lead); writing—review & editing (equal). **Nevena Todorova**: conceptualization (equal); data curation (supporting); formal analysis (supporting); investigation (equal); methodology (equal); supervision (equal); writing—original draft (supporting); writing—review & editing (equal). **Irene Yarovsky**: conceptualization (lead); data curation (supporting); formal analysis (supporting); investigation (equal); methodology (equal); supervision (equal); writing—original draft (supporting); writing—review & editing (equal).
